# Gaussian Guided Self-Adaptive Wolf Search Algorithm Based on Information Entropy Theory

**DOI:** 10.3390/e20010037

**Published:** 2018-01-10

**Authors:** Qun Song, Simon Fong, Suash Deb, Thomas Hanne

**Affiliations:** 1 Department of Computer and Information Science, University of Macau, Macau 999078, China; 2 Decision Sciences and Modelling Program, Victoria University, Melbourne 8001, Australia; 3 Institute for Information Systems, University of Applied Sciences and Arts Northwestern Switzerland, 4600 Olten, Switzerland

**Keywords:** swarm intelligence algorithms, wolf search algorithm, self-adaptation, entropy-guided parameter control

## Abstract

Nowadays, swarm intelligence algorithms are becoming increasingly popular for solving many optimization problems. The Wolf Search Algorithm (WSA) is a contemporary semi-swarm intelligence algorithm designed to solve complex optimization problems and demonstrated its capability especially for large-scale problems. However, it still inherits a common weakness for other swarm intelligence algorithms: that its performance is heavily dependent on the chosen values of the control parameters. In 2016, we published the Self-Adaptive Wolf Search Algorithm (SAWSA), which offers a simple solution to the adaption problem. As a very simple schema, the original SAWSA adaption is based on random guesses, which is unstable and naive. In this paper, based on the SAWSA, we investigate the WSA search behaviour more deeply. A new parameter-guided updater, the Gaussian-guided parameter control mechanism based on information entropy theory, is proposed as an enhancement of the SAWSA. The heuristic updating function is improved. Simulation experiments for the new method denoted as the Gaussian-Guided Self-Adaptive Wolf Search Algorithm (GSAWSA) validate the increased performance of the improved version of WSA in comparison to its standard version and other prevalent swarm algorithms.

## 1. Introduction

In computer science, efficient algorithms for optimizing applications ranging from robot control [1], logistics applications [2] to healthcare management [3] have always evoked great interest. The general aim of an optimization problem is to obtain a solution with a maximum or minimum value to solve the problem. The solution often can be measured as a fitness from a function f(x) where the search space is too huge for a deterministic algorithm to come up with a best solution within a given amount of time [4]. The optimization algorithms are usually either deterministic, of which there are many in operation research, or non-deterministic, which iteratively and stochastically refine a solution using heuristics. For example, in data mining, heuristics-based search algorithms optimize the data clustering efficiency [5] and improve the classification accuracy by feature selection [6]. In the clustering case, different candidate formations of clusters are tried until one is found to be most ideal in terms of the highest similarity among the data in the same cluster. In the classification case, the best feature subset that is most relevant to the prediction target is selected using heuristic means. What these two cases have in common is that the optimization problem is a combinatorial search in nature. The possibilities of choosing a solution in a search space are too huge, which contributes to the NP-hardness of the problem. The search algorithms that are guided by stochastic heuristics are known as meta-heuristics, which literately means a tier of logics controlling the heuristics functions. In this paper, we focus on devising a new meta-heuristic that is parameter-free, based on the semi-swarming type of search algorithms. The swarming kind of search algorithms are contemporary population-based algorithms where search agents form a swarm that moves according to some nature-inspired or biologically-inspired social behavioural patterns. For example, Particle Swarm Optimization (PSO) is the most developed population-based metaheuristic algorithm by which the search agents swarm as a single group during the search operation. Each search particle in PSO has its own velocity, thereby influencing each another; collectively, the agents, which are known as particles, move as one whole large swarm. There are other types of metaheuristics that mimic animal or insect behaviours such as the ant colony algorithm [7] and the firefly algorithm [8] and some new and nature-inspired methods like the water wave algorithm. These algorithms do not always have the agents glued together, moving as one swarm. Instead, the agents move independently, and sometimes, they are scattered. In contrast, these algorithms are known as loosely-packed or semi-swarm bio-inspired algorithms. They have certain advantages in some optimization scenarios. Some well-known semi-swarm algorithms are the Bat Algorithm (BA) [9], the polar bear algorithm [10], the ant lion algorithm, as well as the wolf search algorithm. These algorithms usually embrace search methods that explore the search space both in breath and in depth and mimic swarm movement patterns of animals, insects or even plants found in nature. Their performance in heuristic optimization has been proven to be on par with that of many classical methods including those tight swarm or full swarm algorithms.

However, as optimization problems can be very different from case to case, it is imperative for nature-inspired swarm intelligence algorithms to be adaptive to different situations. The traditional way to solve this kind of problem is to adjust the control parameters manually. This may involve massive trial-and-error to adapt the model behaviour to changing patterns. Once the situation changes, the model may need to be reconfigured for optimal performance. That is why self-adaptive approaches have become more and more attractive for many researchers in recent years.

**Figure 1:**
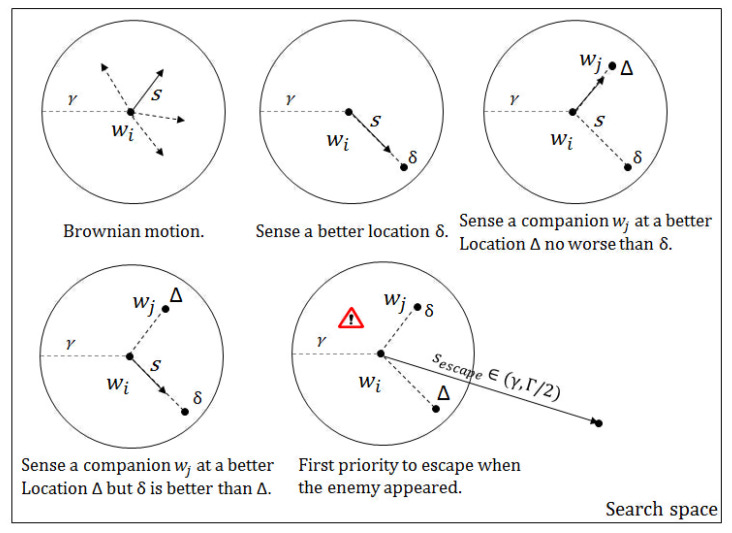
Movement patterns of wolf preying and the algorithm parameters.

Inspired by the preying behaviour of a wolf pack, a contemporary heuristic optimization called the Wolf Search Algorithm (WSA) [11] was proposed. In the wolf swarm, each wolf can not only search for food individually, but they can also merge with their peers when the latter are in a better situation. By this action model, the search can become more efficient as compared to the other single-leader swarms. By mimicking the hunting patterns of a wolf pack, the wolf in WSA as a search agent can find solutions independently, as well as merge with its peers within its visual range. Sometimes, wolves in WSA are simulated to encounter human hunters from whom they will escape to a position far beyond their current one. The human hunters always pose a natural threat to wolves. In the optimization process, this enemy of wolves triggers the search to stay out of local optima and tries out other parts of the search space in the hope of finding better solutions by the algorithm design. As shown in Figure 1, $w_{i}$ is the current search agent (the wolf) and $w_{j}$ is its peer in its visual range γ. Δ and δ are locations in the search agent’s visual range; *S* is the step size of its movement, and Γ is the search space for the objective function. The basic movement of an individual search agent is guided by Brownian motion. In this figure, In most of the metaheuristic algorithms, two of the most popular search methods are Levy search and Brownian search. Levy search is good for exploration [12], and Brownian is efficient for exploiting the optimal solution [13]. In WSA, both search methods were considered. The Brownian motion is used as the basic movement, and the Levy search is for pack movement.

As a typical swarm intelligence heuristic optimization algorithm, WSA shares a common structure and also a common drawback with other algorithms, involving heavy dependence of the efficacy of the algorithm on the chosen parameter values. It is hardly possible to guess the most suitable parameter values for the best algorithm performance. These values are either taken from some suggested defaults or they are manually adjusted. In Figure 1 [14], the parameters’ values remain unchanged during the search operation in the original version of WSA. Quite often, the performance and efficacy of the algorithms for different problems, applications or experimentations would differ greatly, when different parameter values are used. Since there is no golden rule on how the model parameters should be set and the models are sensitive to the parameter values used, users may only guess the values or find the parameter values by trial-and-error. In summary, given that the nature of the swarm search is dynamic, the parameters should be made self-adaptive to the dynamic nature of the problem. Some parameters’ values may be the best at yielding the maximum performance at one time, while other values may be shown to be better in the next moment.

In order to solve this problem, the Self-Adaptive Wolf Search Algorithm (SAWSA) is modified with a combination of techniques, such as a random selection method and a core-guided (or global best-guided) method integrated with the Differential Evolution (DE) crossover function as the parameter-updating mechanism [14]. This SAWSA is based on a randomization, which clearly is not the best option for the rule-based WSA. Compared with the other swarm intelligence algorithms, the most valuable advantage of the original WSA is the stability. However, even though the average results of the published SAWSA are better than those of the WSA, the stability is weakened. To generate a better schema of the algorithm, the implicit relations between the parameters and the performance should be studied. In this paper, we try to find a way to stabilise the performance and generate a self-adaption-guided part for the algorithm. Furthermore, the coding structure is modified. The new algorithm is denoted as the Gaussian-Guided Self-Adaptive Wolf Search Algorithm (GSAWSA).

The contributions of this paper are summarized as follows. Firstly, the self-adaptive parameter range for WSA is carefully improved. Secondly, the parameter updater is not embedded in the main algorithm any longer, and it evolves as an independent updater in this new version. These two changes essentially show different and better optimization performance from the previous version of SAWSA [14]. To verify the performances of the new model once the changes have been made, the experiments are redesigned with settings that enhance the clarity of the result display. The novelty in this paper is the Gaussian-guided parameter updater method. It is a method based on information entropy theory. To improve the performance of SAWSA further, we proposed this novel idea by treating the search agent behaviour as chaotic behaviour, so the algorithm can be perceived as a chaotic system. Using the chaotic stability theory, we can use chaotic maps to guide the operation of the system. Additionally, the entropy value can be used as a measurement of the stability and the inner information communication. In our paper, the feasibility of this new method is analysed, and a suitable map for WSA is found to be a Gaussian map. The advantage of using a Gaussian map as a new method is observed via an extensive simulation experiment.

We verify the efficacy of the considered methods with fourteen typical benchmark functions and compare the performance of GSAWSA with the original WSA, the original bat algorithm and the Hybrid Self-Adaptive Bat Algorithm (HSABA) [15]. The self-adaptiveness is powered by Differential Equations (DE) in SABA. The concept is based on Particle Swarm Optimization (PSO), which moves in some sort of mixed random order and swarming patterns. PSO is one of the classical swarm search algorithms that often shows superior performance with standard benchmark functions. From our investigations, it is supposed that the parameter control by some entropy function in GSAWSA would possibly offer further improvement. The self-adaptive method is a totally hands-free approach that lets the search evolve itself. Parameter control is a guiding approach that steers the course of parameter changes during runtime.

The remainder of the paper is structured as follows: The original wolf search algorithm and the published SAWSA [14] are briefly introduced in Section 2. The chaos system entropy analysis and Gaussian-guided parameter control method are discussed in Section 3, followed by Section 4, which presents the comparison experiments of both the self-adaptive method and the parameter control method with several optional DE functions. The paper ends after presenting concluding remarks in Section 5.

## 2. Related Works and Background

Researchers have extended and improved metaheuristic optimization algorithms to a large extent during the past few decades. The classic algorithms have become more and more mature. Many new and efficient algorithms were invented like those presented in the Introduction. They are tested and shown to be suitable for real case studies. Researchers from other areas have started to use the metaheuristic algorithms in their studies, as well, because of their ease of use. Lately, self-adaptive methods have become popular, and many works in this direction have been published. The purpose of the self-adaptive methods for metaheuristic algorithms is fitting the same algorithm to different problems by self-tuning the parameter values. In the population-based optimization algorithms, two common parameters are very easy to handle: these are the search agents’ population and the search iterations. These two parameters have almost a linear effect with the performance, so the user can choose them judging by the expected level of accuracy and the calculation resources. However, the other parameters are very different from one another in nature. For loosely-packed type of algorithms, one typical research direction is the self-adaptive methods for PSO, the idea being to introduce a check and repair operation to every iterative generation of the search [16]. The idea is suitable for the strong collective swarm algorithms, as well. The self-adaptive firefly algorithm [17] and the hybrid self-adaptive bat algorithm [15] have both proven that the parameter control method makes a great contribution to the search performance. In this paper, as we want to introduce a new parameter control method for WSA, we will first introduce the original WSA and some related self-adaptive methods. Then, this method could be potentially applied to all the other strong collective swarm algorithms.

### 2.1. The Original Wolf Search Algorithm

The WSA is a relatively young, but efficient member of the family of swarm intelligence algorithms. The logic of the WSA search agents is inspired by the hunting behaviour of wolf packs. When preying, wolves use both cooperation and individual work [11]. The wolf applies both local search and a global communication at the same time. To transfer the wolf swarm preying behaviour into a computing method, some basic rules of the WSA are formulated as below.

1. Each wolf search agent has a specific visual range γ, defined by Equation (1).
  (1) $$\gamma \leq d_{\left(w_{i},w_{c}\right)}=(\sum_{k=1}^{n}|w_{i,k}-w_{c,k}{{|}^{\lambda })}^{\frac{1}{\lambda }}$$
  $w_{i}$ is the position of the current search agent; $w_{c}$ is a nearby search agent within visual range γ; and λ is the order of the hyper space.
2. The result of the objective function, which is the fitness value produced from benchmark functions, is used as the measurement of the local position of each search agent (wolf). The search agent can move towards a better location by communication with other agents. Two situations can be found here. One is that the wolf can sense a neighbour with a better location in its visual range. Then, the wolf will move directly towards it. Another situation is that the wolf cannot sense any better peers. Then, the wolf will try to find a better location using a random Brownian movement.
3. To avoid local optima, an escape strategy is introduced to the WSA. An enemy is randomly generated in the search space. If a wolf search agent senses the presence of an enemy, from the current position, it will jump very far away to a new position. The function escape(pa) requires a user input parameter pa, and it generates a new location for an escaped wolf. The function equation is shown in Equation (2) [14].
  (2) $${w_{i}}^{\prime }=w_{i}+\left[rand\cdot \left(\frac{1}{2}\Gamma -\gamma \right)+\gamma \right]\;if\;rand>pa$$
  Γ is the measure of the search space range based on the given upper and lower bounds of the variables, and pa is the escape probability. The behaviour control parameters are listed in Table 1.

**Figure 2:**
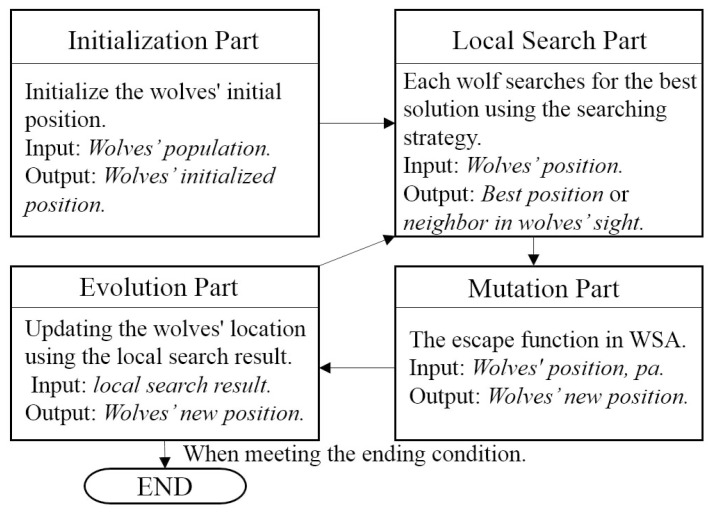
The four main parts of the original Wolf Search Algorithm (WSA).

An example of the wolves’ preying behaviour is illustrated in Figure 1. The original WSA consists of four main parts, which are shown as the four blocks in Figure 2.

The initialization process is important for the original WSA. This is when all the parameters are set with some values. Once the parameters are set, the algorithm proceeds to search for solutions iteratively according to the parameter values, which are fixed throughout the runtime. For all iterative metaheuristics, the parameters control how the iterative search proceeds, such as how the solution evolves and how new candidate solutions are discovered, and the fitter new solutions are replacing the old ones according to some rules coded in the algorithm. For the evolution part, the wolves’ location update function can be summarized by Equation (3).

(3) $${w_{i}}^{\prime }=\left\{\begin{array}{c} \;\;\;\;w_{i}+\alpha \cdot \gamma \cdot rand,\;\;\;\;\;\;\;\;\;\;\;\;\;\;\;\;\mathrm{random}\;\mathrm{movement}\;\;\;\;\;\;\\ w_{i}+\beta_{0}\cdot e^{-\gamma^{2}}\cdot \left(w_{j}-w_{i}\right),\;\;\;w_{j}\;\mathrm{is}\;\mathrm{the}\;\mathrm{result}\;\mathrm{from}\;\mathrm{local}\;\mathrm{search} \end{array}\right.$$

### 2.2. The Self-Adaptive Wolf Search Algorithm

**Figure 3:**
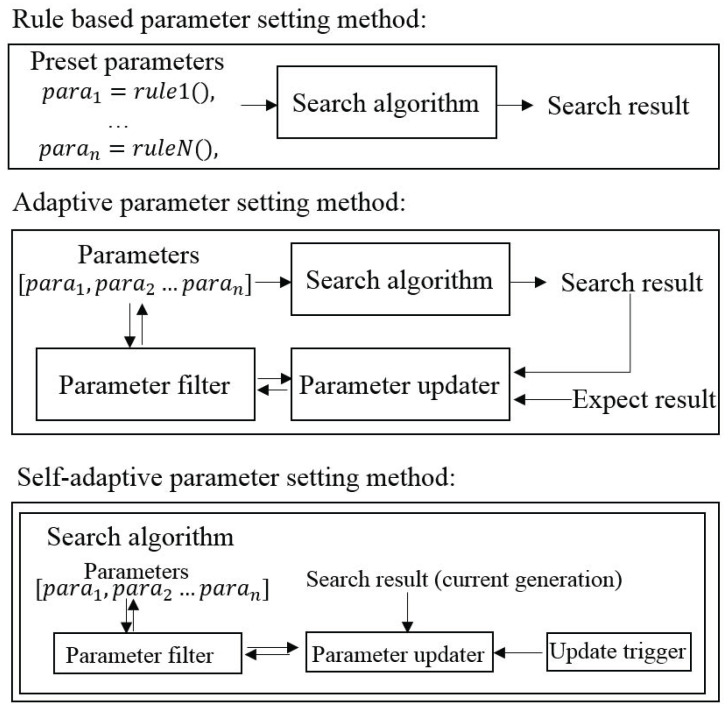
The processing of the self-adaptive method.

As the parameter training is very important for the performance except for the approach based on preset static parameter values, there are three parameter control methods that are usually used. One is the rule-based parameter setting method. The other one is the feedback adaptive method where the parameters are made adaptive to the feedback from the result of the search algorithm [18]. The third approach is the free self-adaptive method, where the parameters can be freely changed during the algorithm run time [15]. Obviously, the adaptive and the self-adaptive methods can be considered as more user friendly, as users do not need to know how to set the parameters or the rules by themselves. From these two methods, the self-adaptive one has turned out to be more popular in the research area, because the search agents’ situation and the local fitness landscape can be dynamically different from generation to generation during the algorithm run. These three methods are conceptually shown in Figure 3.

In 2014, the original bat algorithm is hybridized with a differential evolution strategy called the DE strategy; it was published in [19], known as the Hybrid Self-Adaptive Bat Algorithm (HSABA). The working logics of HSABA are briefly listed as follows:

1. Execute the local search using the DE strategy. A self-adaptation rate, *r*, should be present, which states the ratio of self-adaptive bats to the whole population of bats. In [19], the ratio of 1:10 was used for the number of self-adaptive bats.
2. Choose four virtual bats randomly from the population, each of which was initialized with a new position.
3. Apply the DE strategy to improve the candidate solution.

The WSA was designed for solving complex problems, and the advantage in efficiency becomes more apparent when the search space dimension grows. Distinctive from HSABA, in our SAWSA, the calculation cost is taken into account in the evolution. In contrast to SABA, the SAWSA uses the DE functions instead of embedding the parameters into the search agents. DE functions are a kind of well-developed local search and update function with very low calculation cost.

In the SAWSA, two kinds of self-adaptive methods are used. The first one is called the core-guided one, which is related to the HSABA. During the parameter updating process, HSABA considers the current global best solution from four selected bats using the DE function DE/best/2/bin[15], which is depicted in Equation (4). In this equation, F>0 is a real-valued constant, which adjusts the amplification of the differential variation.

(4) $$b_{j}=bat_{best}+F\cdot \left(bat_{r_{1},j}+bat_{r_{2},j}-bat_{r_{3},j}-bat_{r_{4},j}\right)$$

The other approach is fully random, which is therefore called the random selection DE method, and a simple description is as follows [14]:

1. Randomly select a sufficient number of search agents.
2. Apply the DE functions on the crossover mechanism.
3. Determine if updating the parameters is needed by looking into the current global best solution.
4. Load new values from some allowable range into the parameters.

The pseudocode of the published SAWSA is shown in Algorithm 1. In this algorithm, $w_{i}\left(i=1,...,w\right)$ is the wolf population, $globalfitness_{w_{i}}$ is the global fitness values of each wolf, f(x) is the objective function, the number of parameters is npar (with WSA, the number is four), the control parameters are para[1],...,para[npar] representing γ,s,α,pa, the lower bounds for parameters are $para_{lowerbound}\left[1\right],...,para_{lowerbound}\left[npar\right]$, the upper bounds for parameters are $para_{upperbound}\left[1\right],...,para_{upperbound}\left[npar\right]$, the update probability of parameters is $P_{update}$ and the self-adaptation probability is $P_{self-adapted}$.

**Algorithm 1:** The self-adaptive wolf search algorithm.
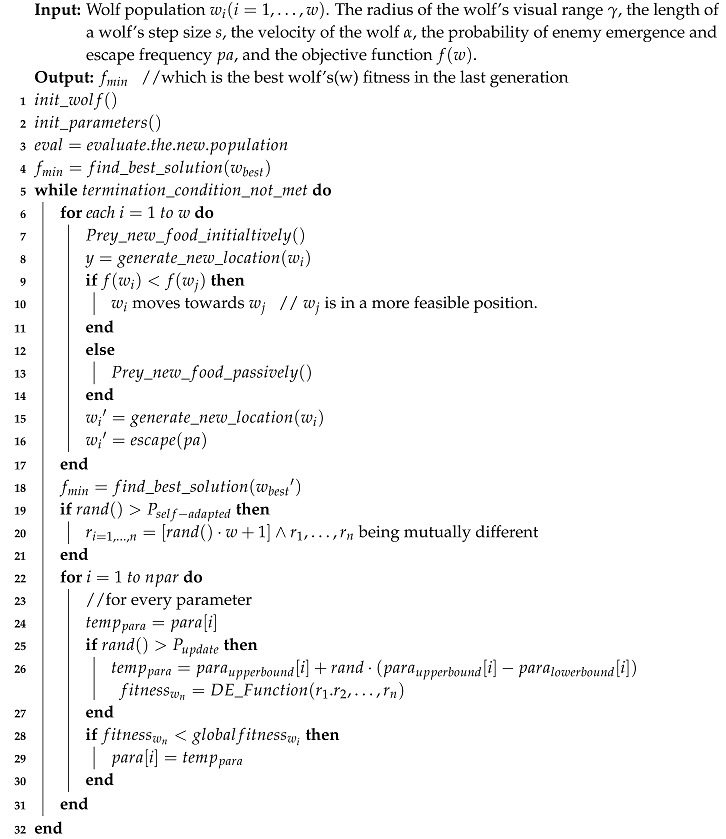


## 3. Gaussian-Guided SAWSA Based on Information Entropy Theory

For the self-adaptive method, the parameter boundaries constitute crucial information [20]. By the parameter definition, an extensive testing was carried out for finding the possible values or ranges for the parameters. Some validated parameter boundaries for SAWSA are shown in Table 2. In the previously-proposed self-adaptive wolf search algorithm, the parameter boundaries are the only limitations of the parameter updating. Clearly, this is not good enough because the ideal parameter control should follow the same patterns such that the changing of the parameter will not affect the algorithm performance.

**Figure 4:**
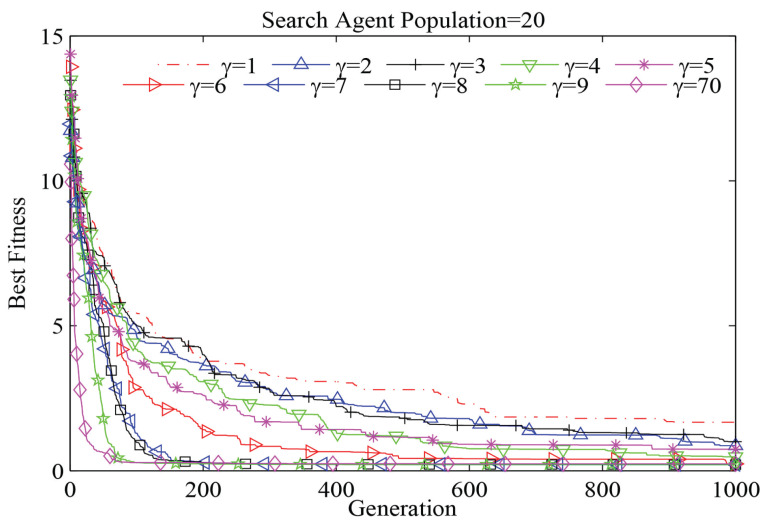
Convergence curves of WSA with γ as the variable.

Just like other classic probabilistic algorithms [21], a mathematical model of WSA is hard to analyse with formal proofs given its stochastic nature. Therefore, we use extensive experiments to gain insights from the experiences with WSA parameter control. To examine the effects of each parameter, the static step size *s*, the velocity factor α, the escape frequency pa and the visual range γ as model variables are used. The results show that already a small change of the parameter limits can obviously affect the performance. When the parameter boundaries are well defined, the performance can hardly be affected. Thereby, it provides consistent optimization performance. In Figure 4, an example with the Ackley 1function for D=2 is shown. Each curve in this figure is the average convergence curve of 20 individuated results (experiment repetitions). Here, the static parameters are s=1, α=0.2 and pa=0.25. The parameter γ changes from one to Γ=70 with a step size of one. Here, as an example, we only evaluate parameter γ. The other parameters follow a similar pattern.

**Figure 5:**
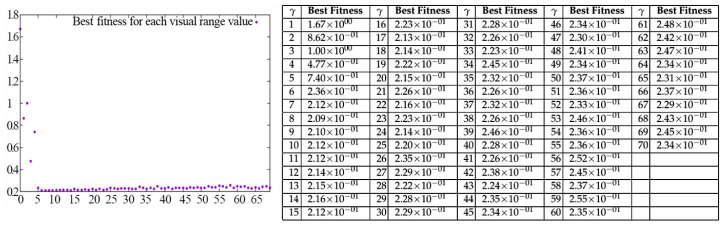
Best fitness value with each γ value.

In Figure 4, most of the 70 curves are overlapping because changing the visual range does not bring much improvement. Only in the range from γ=1 to γ=8, the improvement is obvious. It can be clearly seen that for the Ackley 1function, updating γ in the definitional domain can be used, but is not necessary. The best solution comes from updating γ within a reasonable range where a large improvement can be obtained with the least number of attempts. By analysing the distribution of the best fitness values with the corresponding γ values in Figure 5, we can conclude that the best improvement can be obtained by the approach from the lower bound. This pattern can be shown in our experiments with other benchmark functions, as well. Therefore, for parameter γ, we can give a more reasonable updating domain from the experiments:

(5) 0<γ≤2·ln(Γ)Γ>0

Another pattern can be used as shown in Figure 5, as well, where the best parameter solution is always located in a small range. If we find a sufficiently suitable parameter value, the best way to update is to do so within a certain range, which means the next generated parameter should be guided by the previous one. By using this method, we can avoid unstable performance and save time from random guesses. Subsequently, the challenge now is to choose a suitable entropy function to guide the parameter control approach.

However, as a probability-based algorithm, the current optimization states can influence the results in the next generation, while the future move is highly unpredictable. This model behaviour can be described by the theory of chaos [22], and the parameter update behaviour can be treated as a typical chaotic behaviour; this behaviour seems like a random updating with an uncertain, unrepeatable and unpredictable behaviour in a certain described system [23]. The chaotic phenomenon has to take into account population control, hybrid control or initialized control in metaheuristic algorithm studies [24] and other data science topics [25]. In our study, the entropy theory is used to control the parameter self-update. The reason is analysed as below.

The individual parameter update behaviour is unpredictable by the updater; however, as a chaotic system, the stability can be measured. The chaotic and entropy theory have been used in many schemes [26]. The entropy or entropy rate is one of the efficient measures for the stability of a chaotic system.

The logistic map is one of the most famous chaos maps [27]. It has a polynomial mapping of degree two, which is also denoted as a recurrence relation. Often as an archetypal example, it is cited and used to describe how chaotic and complex a behaviour can become after evolving from some straightforward non-linear dynamical equations. In our paper, the logistic map is used as an example. The map is defined by Equation (6) [28]:

(6) $$f_{\mu }:\;=\left[0,1\right]\to \left[0,1\right]\;\mathrm{given}\;\mathrm{by}\;x_{n+1}=f_{\mu }\left(x_{n}\right),\;\mathrm{where}\;f_{\mu }\left(x\right)=\mu x\left(1-x\right)$$

This map is one of the simplest and most widely-used maps in chaos research, which is also very popular in swarm intelligence. For example, in water engineering, chaos PSO using the logistic map provides a very stable solution to design a flood hydro-graph [29], and it was also used in a resource allocation problem in 2007 [30]. Equation (6) is the mathematic definition of a logistic map; it gives the possible iteration result of a linear problem. To analyse the stability of this map, we can calculate the entropy by using Equation (7).

(7) $$H_{L}=-\sum_{S^{L}\in A}Pr_{S^{L}}log_{2}\left(Pr_{S^{L}}\right)$$

**Figure 6:**
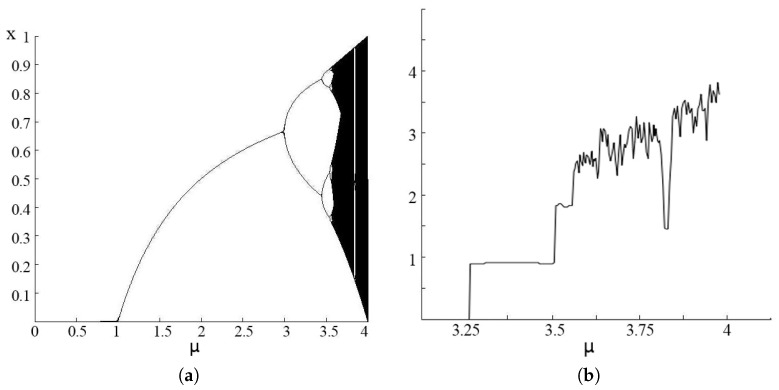
Bifurcation diagram (**a**) and entropy value (**b**) of the logistic map.

The entropy evaluation for the logistic map and the bifurcation diagram is shown in Figure 6. It is clear that the most probable iteration solutions are located near the upper or lower bound of the definition domain. Comparing with the entropy value, the larger the entropy value is, the larger the range of the distribution for the corresponding μ can be provided in the logistic map or defined in information theory as larger information communication. The logistic map can provide a usable way to solve the chaos iteration problem. However, it is not the best way for swarm intelligence, because through many experiments, it is known that the best solution should hardly be located near the boundary. Therefore, here, we consider using another chaos map, which can provide the same stability with a more reasonable iteration outcome.

The Gauss iterated map, which is popularly known as the Gaussian map or mouse map, is another type of a nonlinear one-dimensional iterative map given by the Gaussian function [31] in mathematics:

(8) $$x_{n+1}=exp\left(-\alpha \cdot x_{n}^{2}\right)+\beta$$

With this function, the Gaussian map can be described as the equation:

(9) $$G:ℜ\to ℜ\;\mathrm{defined}\;\mathrm{by}\;G_{\left(x\right)}=e^{-\alpha x^{2}}+\beta$$

where α and β are constant variables. There is a bell-shaped Gaussian function named after Johann Carl Friedrich Gauss. Its shape is similar to the logistic map. As the Gaussian map has two parameters, it can provide more controllability to the iteration outcome, and the G:ℜ→ℜ definition domain can meet more needs than the logistic map. The next step is to choose the most stable and suitable model for use in parameter control. The goal for this step is to satisfy the stability requirement of a chaos system, while keeping up a higher entropy value for more information communication in the system.

The stability of the chaotic maps is defined by the theorem below [32]:

**Theorem** **1.** *Suppose that the map $f_{\mu }\left(x\right)$ has a fixed point at $x^{*}$. Then, the fixed point is stable if:*

(10) $$\left|\frac{d}{d_{x}}f_{\mu }\left(x^{*}\right)\right|<1$$

*and it is unstable if:*

(11) $$\left|\frac{d}{d_{x}}f_{\mu }\left(x^{*}\right)\right|>1$$

The stability analysis of the Gaussian map is shown below.

(12) $$\int G_{\left(x\right)}d_{x}=\int e^{-\alpha x^{2}}+\beta d_{x}=\left|\sqrt{\frac{\pi }{\alpha }}\right|$$

Using Theorem 1, we get the following stability result depending on the parameter values:

(13) $$\left\{\begin{array}{c} x^{*}\;\mathrm{is}\;\mathrm{stable},\;\mathrm{if}\;\sqrt{\frac{\pi }{\alpha }}<1\;\;\;\;\\ x^{*}\;\mathrm{is}\;\mathrm{unstable},\;\mathrm{if}\;\sqrt{\frac{\pi }{\alpha }}>1\; \end{array}\right.$$

**Figure 7:**
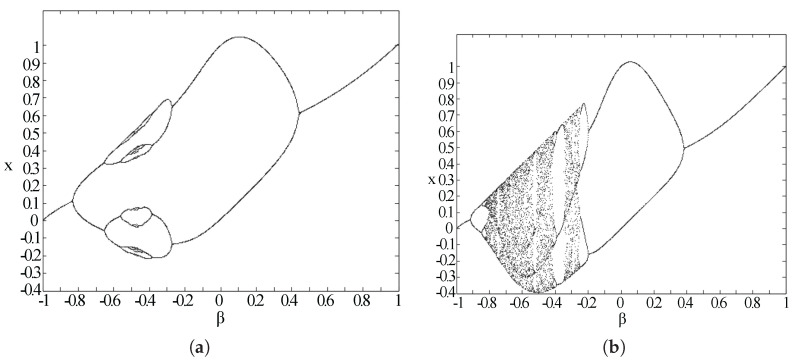
Bifurcation diagram of a Gaussian map when α=4 (**a**) and α=9 (**b**).

**Figure 8:**
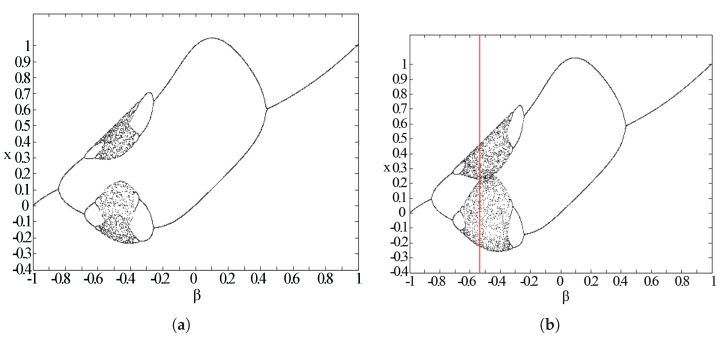
Bifurcation diagram of a Gaussian map when α=5 (**a**) and α=5.4 (**b**).

From the analysis, the stability is just related to α; the Gaussian map will move to stable regions when α>π. In Figure 7, we can see the possible iterative outcome with each β when α=4 and α=9 to visualize the effects of different α values. As shown in Figure 8, this map shows the period-doubling and period-undoubling bifurcations. Considering the parameter iteration needs in our program, based on the entropy theory, the final parameters are set as α=5.4 and β=−0.52, which can provide both stability and enough inner information change.

Using this Gaussian map, the new parameter iterative method will be updated from:

(14) $$temp_{para}=para_{lowerbound}+rand\cdot \left(para_{upperbound}-para_{lowerbound}\right)$$

to:

(15) $$temp_{para}=exp\left(-\alpha \cdot para^{2}\right)+\beta$$

where α and β will be set as above for generating modified parameter values for WSA within the specified boundaries. If this equation leads to negative values in our algorithm, we simply use the absolute value for the parameter update, as all used parameter values should be positive.

Our experiments show that this parameter control method offers more stability and better performance compared to the proposed SAWSA. The parameter control mechanism will be added into the original random selection-based SAWSA, as it has a much better performance than the core-guided DE, which is strongly based on the global best agent (this experiment result is shown in the next section). With this modification, the final version of the Gaussian-guided SAWSA is generated.

To show the advantage of the entropy-based Gaussian-guided parameter update method, which avoids the randomness, we modified all the optional DE functions to self-adaptive methods. By adapting to all these functions, the result can show if the improvement is caused by the adaptive function or the parameter update-guided methods. We mix two different solution selection methods with DE, and the combinations are shown in Table 3. The two self-adaptive types of methods are compared in this paper. Hence, we have a list of differential evolution crossover functions such as DE1, DE2, DE3 functions. In these functions, the current global best solution is taken into consideration. This approach is supposed to increase the stability of the system because the direction of movement is calculated in relation to the location of the current global best. The calculation by RDE1, RDE2, RDE3 and RDE4 is only done at the chosen search agents, so it will not overload the system. The solution by $best_{selected}$ is the one picked as the best current fitness among the other chosen ones. In this way, more randomness can be added to the algorithm by this method. Wider steps are taken for more optional behaviour. So far, it has been tested and worked well with the semi-swarm type of algorithms, such as bat and wolf. It is however not know whether it may become unstable when coupled with other search algorithms.

## 4. Experimental Results

To validate the efficiency of the GSAWSA, we compare it with other well-established and efficient swarm intelligence algorithms such as BA, HSABA and PSO in this paper. In order to prove that the improvement is not caused by the different DE self-adaptive functions, we also use all the optional DE functions in the experiments and select the most suitable function. Afterwards, we test whether the Gaussian-guided method can bring a better performance for the considered problems.

### 4.1. SAWSA Comparative Experiments

The main purpose of this part is to prove that the SAWSA is better than the other algorithms in most cases. Then, we figure out which is the most suitable DE self-adaptive method for the next Gaussian-guided modification. Furthermore, the outcomes of these experiments determine the comparison group of the next experiment. Fourteen standard benchmark functions that are typically used for benchmarking swarm algorithms are used for a credible comparison [33]. They are shown in Table 4.

The objective of the experiments is to compare the suitability of integrating the DE functions, which are listed in Table 3, on various swarm search algorithms, such as SAWSA, WSA, BA, HSABA and PSO. The algorithm combos are benchmarked with those typical standard benchmark functions as appeared in Table 4. The default parameter values used are those suggested from their original papers. An exception is the self-adaptive approaches: there is no parameter value because it is designed to be parameter free. Each benchmark function was tested in various dimensions increasing in complexity from 2, 5, 10, 20, 30 to 50. The population sizes are maintained at two extremes: 20 and 10,000 iterations are repeated for each case. To achieve consistent results, for each function and each dimension, the program was run 50 times for 10,000 cycles, which was how it was done in [34]. Their average curves are then computed, which are shown as the final result.

When the search space dimension is low, all the algorithms can achieve impressive results. The differences are too small to show in both figures and tables. Therefore, for a clearer view, we only present the results for *D* = 30 in Table 5 and the result for *D* = 50 in Table 6. In the result tables, the best results are coloured in bold red, and the second best results are coloured in bold black.

**Figure 9:**
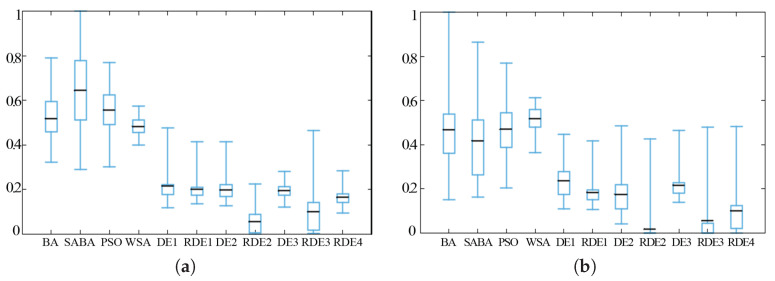
Alpine function (**a**) and Levy 3 test function (**b**) when *D* = 50.

**Figure 10:**
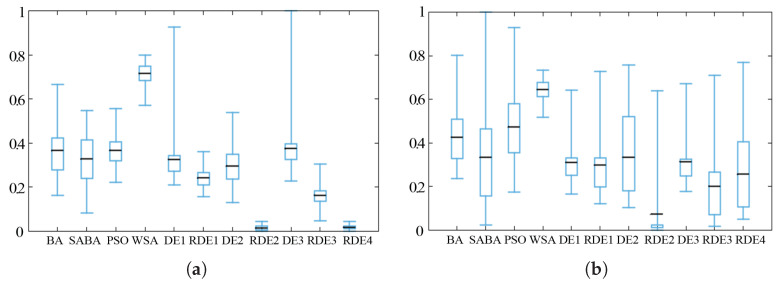
Rastrigin function (**a**) and penalty function (**b**) when *D* = 50.

**Figure 11:**
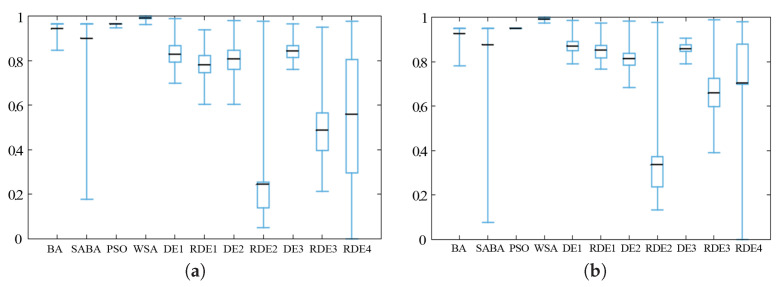
Ackley function when *D* = 30 (**a**) and *D* = 50 (**b**).

Figure 9, Figure 10 and Figure 11 show the box-plots of some standard benchmark functions’ comparisons for the same or different dimensions. The black line in the middle of the box-plots indicates the average baseline; half of the result range is shown by the size of the inner box. For easy visual comparison on the same axis, the ranges for all the data have been normalized.

### 4.2. Gaussian-Guided Parameter Control Comparative Experiments

In this section, the experiment purpose is to prove the efficiency of the entropy-guided parameter control mechanism using the Gaussian function for a substantiation of the expected benefits of SAWSA. From the previous section, we can see clearly that the random selection method is more suitable as a self-adaptive method for WSA. Therefore, in this experiment, to reduce the redundancy, we only add the entropy-guided parameter control mechanism using the Gaussian function to the randomization part of the DE self-adaptive WSA with the four functions listed in Table 3. They are referred to as RDE1, RDE2, RDE3 and RDE4. For comparison, the entropy-guided SAWSA in the searching part is generated using the Gaussian function, and they referred to as GRDE1, GRDE2, GRDE3 and GRDE4 in this experiment. We also use the 14 benchmark functions from Table 4, which are tested for the dimensions 2, 5, 10, 20, 30 and 50. The maximum number of generations and the population size are set as gen= 10,000 and static pop=20, respectively. For consistent results and a fair comparison, each case was run 50 times, and we use the average for the final data analysis.

When the dimension is low, all algorithms have a similar performance, and the algorithmic enhancement seems unnecessary. However, when *D* increases to a large number (which means the objective function is very complex), the enhancement provided by this entropy-based Gaussian-guided parameter control method can be clearly observed. Therefore, here, we only show the experiment results for D=30 and D=50 in Table 7 and Table 8. The best results are marked in red.

**Figure 12:**
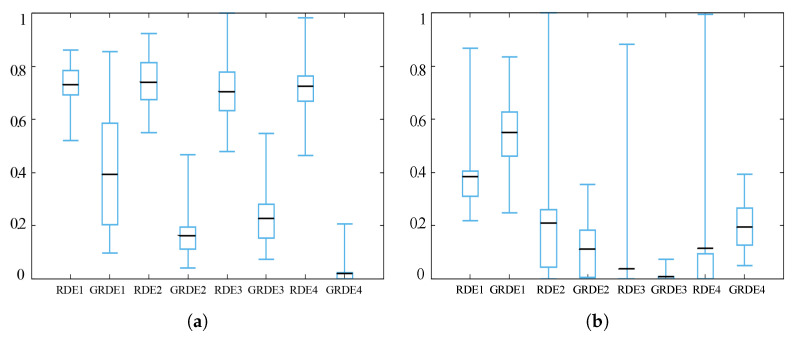
Deb 1 function (**a**) and Levy 3 test function (**b**) when *D* = 50.

**Figure 13:**
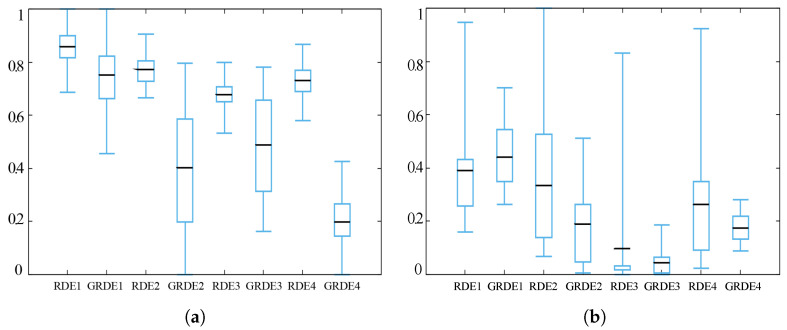
Michalewicz test function (**a**) and Penalty 1 function (**b**) when *D* = 50.

In Table 7 and Table 8, we can see that most of the best results are obtained by the entropy-based Gaussian-guided SAWSA. The entropy-based Gaussian-guided methods not only enhance the performance, but also stabilize it. In the box charts as shown in Figure 12 and Figure 13, the boxes show the location ranges of middle half of the result data, while the black lines show the average data. For better visual comparison, all the data are normalized. In most cases, the entropy-guided SAWSA produces better performance than the original SAWSA, and even every entropy-guided algorithm has a much smaller box in the box chart than the SAWSA comparison algorithm. For a metaheuristic algorithm. this improvement is significant, as stability is a very important attribute, which is often very hard to achieve.

For the statistical tests, we use the *p*-values as the measurement and the WSA as the control method. Due to page limitation, we cannot show all the comparisons with other control methods. Therefore, we focus on improving the original WSA. The hand *p*-values are shown in Table 9. Knowing the “no free lunch” policy [35], the improvement resulted in the algorithm costing more calculation resources than the original WSA. In Table 10, the CPU times are listed for all the algorithms with D=30 and D=50. The CPU time is defined as the total run time for each algorithm that runs 1000 generations with 20 search agents.

## 5. Conclusions

Self-adaptive methods are effective to enable parameter-free metaheuristic algorithms; they can even improve the performance because the parameters’ values are self-tuned to be optimal (hence optimal results). Based on the authors’ prior work about the hybridizing self-adaptive DE functions in the bat algorithm, a similar, but new self-adaptive algorithm was developed called SAWSA. However, due to the lack of stability control and missing knowledge of the inner connection of the parameters and performance, the SAWSA is not sufficiently satisfactory for us. In this paper, the self-adaptive method is considered from the perspective of entropy theory for metaheuristic algorithms, and we developed an improved parameter control called the Gaussian-guided self-adaptive method. After designing this method denoted as GSAWSA, we configured test experiments with fourteen standard benchmark functions. Based on the results, the following conclusions can be drawn.

Firstly, the self-adaptive approach is proven to be very efficient for metaheuristic algorithms, especially those that require much calculation cost. By using this method, the parameter training part can be removed in the real case usage. However, as the self-adaptive modification would increase the complexity of the algorithm, the calculation cost of the self-adaptive method must be taken into consideration.

Secondly, comparing all the optional self-adaptive DE methods in the experiment, the type of random selection is a better choice for SAWSA. However, as the average outcome is improved, the stability is clearly decreased by introducing more randomness into the algorithm. How to balance the random effects and to improve the stability is, in general, a difficult challenge for a the metaheuristic algorithm study.

Thirdly, the parameter-performance changing pattern can be considered a very important feature of metaheuristic algorithms. Normally, researchers use the performance or the situation feedback as an update reference. However, how the parameters influence the performance usually remains outside of consideration. By analysing how the parameters influence the performance, a better self-adaptive updating method could be developed and a better performance could be achieved with less computing resources.

In conclusion, a parameter-free metaheuristic model where a Gaussian map is fused with the WSA algorithm is proposed. It has the advantages of not requiring the parameters’ values to remain static and the parameters will tune themselves as the search operation proceeds. Often, from our experiment results, the new model shows improvement in performance compared to the naive version of WSA, as well as other similar swarm algorithms. As future work, we want to investigate in depth the computational analysis of how the Gaussian map contributes to refining the performance and preventing the search from converging prematurely to local optima. The analysis should be done together with the runtime cost, as well. It is known from the experiments reported in this paper that there is a certain overhead when the Gaussian map is fused with WSA, extending its performance, but at the same time, the extra computation consumes additional resources. In the future the GAWSA should be enhanced with the capability of balancing the runtime cost and the best possible performance in terms of the solution quality obtained.

## Figures and Tables

**Table 1:** Behaviour control parameters.

Parameter	Definition
γ	The visual radius of a wolf agent
*s*	The step size of a wolf agent
α	The velocity of the wolf agent
pa	The probability of having enemy presence

**Table 2:** Boundaries of behaviour control parameters.

Parameter	Update Range	Definition
γ	(0, 2·ln(Γ)] by experiment	visual range
*s*	(0, 1] by definition	step size
α	(0, 1] by both experiment and definition	velocity factor
pa	[0, 1] by definition	escape probability

**Table 3:** The names of Differential Evolution (DE) functions that are implemented with various solution selection methods.

Function Name	DE Function
Core-guided DE method	
*DE*1	$\hat{w}=w_{globalbest}+F\cdot \left(w_{r_{1},j}+w_{r_{2},j}-w_{r_{3},j}-w_{r_{4},j}\right)$
*DE*2	$\hat{w}=w_{r_{1},j}+F\cdot \left(w_{globalbest}-w_{r_{2},j}\right)-F\cdot \left(w_{r_{3},j}-w_{r_{4},j}\right)$
*DE*3	$\hat{w}=w_{globalbest}+F\cdot \left(w_{r_{1},j}-w_{r_{2},j}\right)$
Random selection DE method	
*RDE*1	$\hat{w}=w_{selectedbest}+F\cdot \left(w_{r_{1},j}+w_{r_{2},j}-w_{r_{3},j}-w_{r_{4},j}\right)$
*RDE*2	$\hat{w}=w_{r_{1},j}+F\cdot \left(w_{selectedbest}-w_{r_{2},j}\right)-F\cdot \left(w_{r_{3},j}-w_{r_{4},j}\right)$
*RDE*3	$\hat{w}=w_{selectedbest}+F\cdot \left(w_{r_{1},j}-w_{r_{2},j}\right)$
*RDE*4	$\hat{w}=w_{r_{1},j}+F\cdot \left(w_{r_{2},j}-w_{r_{3},j}\right)$

**Table 4:** Standard benchmark functions.

*f*	Function Name	Search Range	Global Best
$f_{1}$	Ackley function	[−35, 35]	$f(x^{*})=0$
$f_{2}$	Alpine function	[−10, 10]	$f(x^{*})=0$
$f_{3}$	Csendes function	[−1, 1]	$f(x^{*})=0$
$f_{4}$	Deb 1 function	[−1, 1]	$f(x^{*})=0$
$f_{5}$	Deflected Corrugated Spring function	[0, 10]	$f(x^{*})=0$
$f_{6}$	Dixon and Price function	[−10, 10]	$f(x^{*})=0$
$f_{7}$	Infinity test function	[−1, 1]	$f(x^{*})=0$
$f_{8}$	Levy 3 test function	[−10, 10]	$f(x^{*})=0$
$f_{9}$	Michalewicz test function	[0, pi]	$f(x^{*})=-1.8013$
$f_{10}$	Mishra 7 test function	[−10, 10]	$f(x^{*})=0$
$f_{11}$	Moved axis function	[−5.12, 5.12]	$f(x^{*})=0$
$f_{12}$	Penalty 1 function	[−50, 50]	$f(x^{*})=0$
$f_{13}$	Rastrigin function	[−15, 15]	$f(x^{*})=0$
$f_{14}$	Rosenbrock function	[−15, 15]	$f(x^{*})=0$

**Table 5:** Result data when *D* = 30. HSABA, Hybrid Self-Adaptive Bat Algorithm; SAWSA, Self-Adaptive Wolf Search Algorithm.

Fun. ^a^	Meas. ^b^	PSO	BA	HSABA	WSA	SAWSA						
						*DE*1	*DE*2	*DE*3	*RDE*1	*RDE*2	*RDE*3	*RDE*4
**f1**	**Aver. ^c^**	2.00$\times 10^{01}$	1.89$\times 10^{01}$	2.00$\times 10^{01}$	2.02$\times 10^{01}$	1.36$\times 10^{01}$	1.22$\times 10^{01}$	1.45$\times 10^{01}$	9.30$\times 10^{00}$	**3.60$\times 10^{00}$**	1.00$\times 10^{01}$	**7.06$\times 10^{-01}$**
	**Stdev. ^d^**	**5.58$\times 10^{-07}$**	1.98$\times 10^{00}$	**1.31$\times 10^{-06}$**	5.30$\times 10^{-02}$	8.32$\times 10^{-01}$	5.90$\times 10^{00}$	1.96$\times 10^{00}$	9.93$\times 10^{-01}$	1.26$\times 10^{00}$	6.99$\times 10^{00}$	5.71$\times 10^{-01}$
**f2**	**Aver.**	2.30$\times 10^{01}$	7.59$\times 10^{00}$	3.75$\times 10^{01}$	1.51$\times 10^{01}$	7.99$\times 10^{-01}$	6.85$\times 10^{-01}$	2.15$\times 10^{00}$	6.09$\times 10^{-01}$	**1.99$\times 10^{-06}$**	3.46$\times 10^{-03}$	**5.34$\times 10^{-04}$**
	**Stdev.**	3.13$\times 10^{01}$	1.65$\times 10^{01}$	1.00$\times 10^{02}$	2.40$\times 10^{00}$	2.37$\times 10^{-02}$	1.89$\times 10^{-02}$	1.07$\times 10^{01}$	8.95$\times 10^{-03}$	**7.90$\times 10^{-11}$**	1.75$\times 10^{-04}$	**1.79$\times 10^{-06}$**
**f3**	**Aver.**	6.40$\times 10^{-03}$	3.94$\times 10^{-06}$	5.42$\times 10^{-01}$	1.62$\times 10^{-03}$	2.55$\times 10^{-11}$	2.34$\times 10^{-11}$	3.43$\times 10^{-12}$	2.65$\times 10^{-11}$	**6.07$\times 10^{-13}$**	3.14$\times 10^{-12}$	**2.51$\times 10^{-14}$**
	**Stdev.**	1.07$\times 10^{-04}$	1.40$\times 10^{-12}$	3.66$\times 10^{00}$	1.23$\times 10^{-06}$	1.40$\times 10^{-22}$	7.88$\times 10^{-23}$	1.31$\times 10^{-23}$	9.01$\times 10^{-23}$	**9.31$\times 10^{-25}$**	2.88$\times 10^{-23}$	**7.44$\times 10^{-27}$**
**f4**	**Aver.**	−7.77 $\times 10^{-01}$	−9.03 $\times 10^{-01}$	−4.64 $\times 10^{-01}$	−6.35 $\times 10^{-01}$	−9.96 $\times 10^{-01}$	−9.96 $\times 10^{-01}$	−9.97 $\times 10^{-01}$	−9.96 $\times 10^{-01}$	−9.97 $\times 10^{-01}$	−9.97 $\times 10^{-01}$	−9.97 $\times 10^{-01}$
	**Stdev.**	2.21$\times 10^{-03}$	3.49$\times 10^{-03}$	1.12$\times 10^{-03}$	1.98$\times 10^{-04}$	**3.52$\times 10^{-07}$**	1.37$\times 10^{-06}$	**4.50$\times 10^{-07}$**	4.59$\times 10^{-07}$	7.05$\times 10^{-07}$	4.85$\times 10^{-07}$	6.90$\times 10^{-07}$
**f5**	**Aver.**	5.07$\times 10^{00}$	7.11$\times 10^{00}$	1.29$\times 10^{01}$	1.46$\times 10^{01}$	3.47$\times 10^{00}$	2.83$\times 10^{00}$	5.06$\times 10^{00}$	2.37$\times 10^{00}$	−1.17 $\times 10^{00}$	9.22$\times 10^{-01}$	−1.84 $\times 10^{00}$
	**Stdev.**	8.68$\times 10^{00}$	1.32$\times 10^{01}$	3.34$\times 10^{01}$	5.15$\times 10^{00}$	3.01$\times 10^{00}$	4.61$\times 10^{00}$	4.09$\times 10^{00}$	1.50$\times 10^{00}$	**5.67$\times 10^{-01}$**	2.35$\times 10^{00}$	**2.54$\times 10^{-01}$**
**f6**	**Aver.**	1.17$\times 10^{04}$	**3.98$\times 10^{-01}$**	2.94$\times 10^{05}$	1.16$\times 10^{01}$	7.75$\times 10^{-01}$	1.56$\times 10^{00}$	1.83$\times 10^{00}$	5.86$\times 10^{-01}$	**2.65$\times 10^{-01}$**	7.45$\times 10^{-01}$	1.19$\times 10^{00}$
	**Stdev.**	4.71$\times 10^{08}$	**1.73$\times 10^{-01}$**	1.35$\times 10^{11}$	8.53$\times 10^{00}$	3.54$\times 10^{-01}$	6.90$\times 10^{00}$	3.30$\times 10^{00}$	2.79$\times 10^{-01}$	**6.92$\times 10^{-05}$**	8.33$\times 10^{-01}$	9.57$\times 10^{-01}$
**f7**	**Aver.**	1.36$\times 10^{-02}$	3.46$\times 10^{-06}$	2.58$\times 10^{-01}$	1.73$\times 10^{-03}$	3.48$\times 10^{-11}$	2.16$\times 10^{-11}$	2.71$\times 10^{-12}$	2.85$\times 10^{-11}$	**7.01$\times 10^{-13}$**	2.31$\times 10^{-12}$	**1.93$\times 10^{-14}$**
	**Stdev.**	2.30$\times 10^{-04}$	1.27$\times 10^{-12}$	4.96$\times 10^{-01}$	1.24$\times 10^{-06}$	2.47$\times 10^{-22}$	1.22$\times 10^{-22}$	9.58$\times 10^{-24}$	1.73$\times 10^{-22}$	**1.73$\times 10^{-24}$**	1.20$\times 10^{-23}$	**2.81$\times 10^{-27}$**
**f8**	**Aver.**	3.64$\times 10^{01}$	3.22$\times 10^{00}$	5.17$\times 10^{01}$	4.48$\times 10^{01}$	1.40$\times 10^{-04}$	9.06$\times 10^{-02}$	1.69$\times 10^{00}$	**9.88$\times 10^{-05}$**	9.05$\times 10^{-02}$	6.63$\times 10^{-01}$	**1.39$\times 10^{-07}$**
	**Stdev.**	3.56$\times 10^{02}$	1.97$\times 10^{01}$	1.01$\times 10^{03}$	4.52$\times 10^{01}$	1.32$\times 10^{-08}$	7.77$\times 10^{-02}$	6.65$\times 10^{00}$	**2.99$\times 10^{-10}$**	7.77$\times 10^{-02}$	1.06$\times 10^{00}$	**3.69$\times 10^{-13}$**
**f9**	**Aver.**	−1.26 $\times 10^{01}$	−1.41 $\times 10^{01}$	−1.60 $\times 10^{01}$	−1.08 $\times 10^{01}$	−2.47 $\times 10^{01}$	−2.66 $\times 10^{01}$	−2.87 $\times 10^{01}$	−2.59 $\times 10^{01}$	−2.95 $\times 10^{01}$	−2.92$\times 10^{01}$	−2.96 $\times 10^{01}$
	**Stdev.**	1.09$\times 10^{00}$	4.71$\times 10^{00}$	5.19$\times 10^{00}$	3.56$\times 10^{-01}$	4.64$\times 10^{-01}$	1.60$\times 10^{-01}$	2.36$\times 10^{-01}$	3.48$\times 10^{-01}$	**9.69$\times 10^{-03}$**	9.18$\times 10^{-02}$	**1.09$\times 10^{-03}$**
**f10**	**Aver.**	6.98$\times 10^{64}$	**1.34$\times 10^{49}$**	6.98$\times 10^{64}$	9.67$\times 10^{52}$	3.51$\times 10^{51}$	3.27$\times 10^{51}$	3.34$\times 10^{51}$	4.60$\times 10^{51}$	**3.18$\times 10^{51}$**	8.05$\times 10^{51}$	5.61$\times 10^{51}$
	**Stdev.**	**5.76$\times 10^{98}$**	**5.59$\times 10^{98}$**	**5.76$\times 10^{98}$**	2.74$\times 10^{106}$	2.50$\times 10^{103}$	3.97$\times 10^{103}$	6.43$\times 10^{103}$	4.22$\times 10^{103}$	1.79$\times 10^{103}$	3.81$\times 10^{104}$	1.18$\times 10^{104}$
**f11**	**Aver.**	1.83$\times 10^{03}$	9.93$\times 10^{-04}$	2.89$\times 10^{03}$	2.89$\times 10^{01}$	1.69$\times 10^{-01}$	1.85$\times 10^{-01}$	7.29$\times 10^{-01}$	1.40$\times 10^{-01}$	**2.77$\times 10^{-30}$**	**1.65$\times 10^{-13}$**	7.80$\times 10^{-05}$
	**Stdev.**	7.90$\times 10^{05}$	5.36$\times 10^{-08}$	5.45$\times 10^{06}$	2.62$\times 10^{02}$	1.11$\times 10^{-02}$	8.92$\times 10^{-03}$	9.67$\times 10^{-02}$	2.16$\times 10^{-03}$	**6.80$\times 10^{-59}$**	**5.47$\times 10^{-25}$**	7.53$\times 10^{-08}$
**f12**	**Aver.**	8.98$\times 10^{01}$	7.04$\times 10^{01}$	2.21$\times 10^{02}$	1.32$\times 10^{02}$	1.84$\times 10^{01}$	7.16$\times 10^{00}$	2.51$\times 10^{01}$	5.04$\times 10^{00}$	3.63$\times 10^{-02}$	7.39$\times 10^{-01}$	**9.88$\times 10^{-09}$**
	**Stdev.**	2.16$\times 10^{03}$	1.66$\times 10^{03}$	1.84$\times 10^{04}$	1.21$\times 10^{02}$	1.75$\times 10^{02}$	5.01$\times 10^{01}$	6.93$\times 10^{02}$	3.34$\times 10^{00}$	**1.05$\times 10^{-02}$**	3.57$\times 10^{00}$	**6.32$\times 10^{-16}$**
**f13**	**Aver.**	5.42$\times 10^{02}$	8.60$\times 10^{02}$	4.26$\times 10^{02}$	9.62$\times 10^{02}$	2.40$\times 10^{02}$	3.00$\times 10^{02}$	5.75$\times 10^{02}$	1.35$\times 10^{02}$	4.48$\times 10^{-01}$	2.11$\times 10^{02}$	**5.00$\times 10^{-02}$**
	**Stdev.**	1.80$\times 10^{04}$	1.05$\times 10^{05}$	6.20$\times 10^{04}$	1.04$\times 10^{04}$	5.94$\times 10^{03}$	2.27$\times 10^{04}$	2.01$\times 10^{04}$	2.17$\times 10^{03}$	**1.40$\times 10^{00}$**	1.21$\times 10^{04}$	**4.97$\times 10^{-02}$**
**f14**	**Aver.**	1.26$\times 10^{05}$	8.07$\times 10^{01}$	2.20$\times 10^{06}$	7.50$\times 10^{01}$	**1.10$\times 10^{01}$**	8.16$\times 10^{01}$	1.15$\times 10^{02}$	1.83$\times 10^{01}$	4.25$\times 10^{01}$	2.07$\times 10^{01}$	5.01$\times 10^{01}$
	**Stdev.**	2.09$\times 10^{10}$	8.05$\times 10^{03}$	1.59$\times 10^{13}$	**1.18$\times 10^{02}$**	7.95$\times 10^{02}$	1.80$\times 10^{04}$	1.58$\times 10^{04}$	6.48$\times 10^{02}$	1.45$\times 10^{03}$	7.11$\times 10^{02}$	2.26$\times 10^{03}$

^a^ Short for Function, listed the benchmark function number. ^b^ Short for Measures, in this table two measures are used, the average number and the standard deviation. ^c^ The average number of the best fitness result set. ^d^ The standard deviation of the best fitness result set.

**Table 6:** Result data when *D* = 50.

Fun.	Meas.	PSO	BA	HSABA	WSA	SAWSA						
						*DE*1	*DE*2	*DE*3	*RDE*1	*RDE*2	*RDE*3	*RDE*4
**f1**	**Aver.**	2.00$\times 10^{01}$	1.89$\times 10^{01}$	2.05$\times 10^{01}$	2.00$\times 10^{01}$	1.49$\times 10^{01}$	1.27$\times 10^{01}$	1.49$\times 10^{01}$	1.20$\times 10^{01}$	**1.32$\times 10^{00}$**	1.22$\times 10^{01}$	**6.21$\times 10^{00}$**
	**Stdev.**	**6.80$\times 10^{-07}$**	1.22$\times 10^{00}$	8.55$\times 10^{-03}$	**3.49$\times 10^{-07}$**	7.00$\times 10^{-01}$	4.90$\times 10^{00}$	3.15$\times 10^{00}$	4.93$\times 10^{-01}$	8.02$\times 10^{-01}$	3.62$\times 10^{00}$	4.56$\times 10^{00}$
**f2**	**Aver.**	4.41$\times 10^{01}$	1.73$\times 10^{01}$	3.82$\times 10^{01}$	6.07$\times 10^{01}$	2.89$\times 10^{00}$	2.44$\times 10^{00}$	6.80$\times 10^{00}$	2.59$\times 10^{00}$	**4.93$\times 10^{-02}$**	2.67$\times 10^{-01}$	**4.27$\times 10^{-02}$**
	**Stdev.**	1.23$\times 10^{02}$	6.32$\times 10^{01}$	4.94$\times 10^{00}$	3.26$\times 10^{02}$	2.16$\times 10^{-01}$	2.83$\times 10^{-01}$	8.84$\times 10^{01}$	1.01$\times 10^{00}$	**9.06$\times 10^{-03}$**	3.18$\times 10^{-01}$	**3.50$\times 10^{-02}$**
**f3**	**Aver.**	9.57$\times 10^{-02}$	4.67$\times 10^{-06}$	2.36$\times 10^{-03}$	1.45$\times 10^{00}$	1.15$\times 10^{-09}$	8.21$\times 10^{-10}$	**1.92$\times 10^{-10}$**	1.08$\times 10^{-09}$	**1.69$\times 10^{-12}$**	9.25$\times 10^{-10}$	3.17$\times 10^{-10}$
	**Stdev.**	6.83$\times 10^{-02}$	1.64$\times 10^{-12}$	5.92$\times 10^{-06}$	4.51$\times 10^{00}$	1.99$\times 10^{-19}$	1.09$\times 10^{-19}$	**3.92$\times 10^{-20}$**	2.86$\times 10^{-19}$	**6.04$\times 10^{-24}$**	6.34$\times 10^{-18}$	4.34$\times 10^{-19}$
**f4**	**Aver.**	−6.63 $\times 10^{-01}$	−9.12 $\times 10^{-01}$	−5.60 $\times 10^{-01}$	−4.45 $\times 10^{-01}$	−9.87 $\times 10^{-01}$	−9.87 $\times 10^{-01}$	−9.88 $\times 10^{-01}$	−9.88 $\times 10^{-01}$	−9.89 $\times 10^{-01}$	−9.90 $\times 10^{-01}$	−9.89 $\times 10^{-01}$
	**Stdev.**	2.46$\times 10^{-03}$	2.05$\times 10^{-03}$	6.75$\times 10^{-05}$	1.22$\times 10^{-03}$	**2.13$\times 10^{-06}$**	5.99$\times 10^{-06}$	**1.86$\times 10^{-06}$**	4.22$\times 10^{-06}$	2.76$\times 10^{-06}$	3.79$\times 10^{-06}$	2.61$\times 10^{-06}$
**f5**	**Aver.**	8.03$\times 10^{00}$	1.31$\times 10^{01}$	2.71$\times 10^{01}$	2.17$\times 10^{01}$	1.02$\times 10^{01}$	8.98$\times 10^{00}$	1.31$\times 10^{01}$	7.87$\times 10^{00}$	−1.89 $\times 10^{00}$	3.73$\times 10^{00}$	−5.62 $\times 10^{-01}$
	**Stdev.**	6.65$\times 10^{00}$	2.85$\times 10^{01}$	6.14$\times 10^{00}$	3.03$\times 10^{01}$	3.65$\times 10^{00}$	3.08$\times 10^{00}$	1.28$\times 10^{01}$	4.83$\times 10^{00}$	**8.01$\times 10^{-01}$**	8.86$\times 10^{00}$	**1.59$\times 10^{00}$**
**f6**	**Aver.**	3.11$\times 10^{05}$	1.63$\times 10^{00}$	3.80$\times 10^{01}$	9.00$\times 10^{05}$	**1.16$\times 10^{00}$**	2.07$\times 10^{00}$	3.26$\times 10^{00}$	1.42$\times 10^{00}$	6.61$\times 10^{00}$	1.48$\times 10^{00}$	**6.69$\times 10^{-01}$**
	**Stdev.**	1.57$\times 10^{11}$	8.64$\times 10^{00}$	1.37$\times 10^{02}$	7.01$\times 10^{11}$	**2.13$\times 10^{-01}$**	3.44$\times 10^{00}$	9.54$\times 10^{00}$	**4.32$\times 10^{-01}$**	1.93$\times 10^{01}$	2.52$\times 10^{00}$	6.77$\times 10^{-01}$
**f7**	**Aver.**	3.03$\times 10^{-02}$	4.37$\times 10^{-06}$	2.08$\times 10^{-03}$	1.25$\times 10^{00}$	1.24$\times 10^{-09}$	7.58$\times 10^{-10}$	**1.61$\times 10^{-10}$**	1.21$\times 10^{-09}$	**1.32$\times 10^{-12}$**	1.97$\times 10^{-10}$	2.13$\times 10^{-09}$
	**Stdev.**	2.09$\times 10^{-03}$	3.45$\times 10^{-12}$	1.52$\times 10^{-06}$	8.03$\times 10^{00}$	2.91$\times 10^{-19}$	9.67$\times 10^{-20}$	**4.87$\times 10^{-20}$**	3.46$\times 10^{-19}$	**3.08$\times 10^{-24}$**	2.66$\times 10^{-19}$	8.12$\times 10^{-17}$
**f8**	**Aver.**	7.84$\times 10^{01}$	5.70$\times 10^{00}$	8.59$\times 10^{01}$	1.07$\times 10^{02}$	1.11$\times 10^{00}$	1.40$\times 10^{-01}$	1.52$\times 10^{00}$	**1.36$\times 10^{-01}$**	**8.59$\times 10^{-07}$**	5.68$\times 10^{-01}$	1.85$\times 10^{-01}$
	**Stdev.**	8.69$\times 10^{02}$	4.69$\times 10^{01}$	8.78$\times 10^{01}$	2.81$\times 10^{03}$	6.72$\times 10^{00}$	**2.12$\times 10^{-01}$**	6.45$\times 10^{00}$	3.67$\times 10^{-01}$	**1.65$\times 10^{-12}$**	1.82$\times 10^{00}$	2.39$\times 10^{-01}$
**f9**	**Aver.**	−1.75 $\times 10^{01}$	−2.00 $\times 10^{01}$	−1.41 $\times 10^{01}$	−2.63 $\times 10^{01}$	−3.84 $\times 10^{01}$	−4.09 $\times 10^{01}$	−4.69 $\times 10^{01}$	−3.94 $\times 10^{01}$	−4.71 $\times 10^{01}$	−4.81 $\times 10^{01}$	−4.67 $\times 10^{01}$
	**Stdev.**	4.10$\times 10^{00}$	1.01$\times 10^{01}$	2.97$\times 10^{-01}$	2.65$\times 10^{01}$	9.17$\times 10^{-01}$	6.45$\times 10^{-01}$	7.22$\times 10^{-01}$	5.30$\times 10^{-01}$	**1.90$\times 10^{-01}$**	**2.11$\times 10^{-01}$**	2.46$\times 10^{-01}$
**f10**	**Aver.**	9.25$\times 10^{128}$	9.94$\times 10^{128}$	1.01$\times 10^{117}$	9.55$\times 10^{119}$	8.11$\times 10^{115}$	5.04$\times 10^{115}$	1.09$\times 10^{116}$	7.82$\times 10^{115}$	**3.60$\times 10^{115}$**	**3.47$\times 10^{115}$**	1.07$\times 10^{116}$
	**Stdev.**	9.97$\times 10^{226}$	9.97$\times 10^{226}$	4.10$\times 10^{226}$	9.97$\times 10^{226}$	**1.71$\times 10^{226}$**	**1.25$\times 10^{226}$**	4.52$\times 10^{226}$	2.19$\times 10^{226}$	4.84$\times 10^{226}$	2.19$\times 10^{226}$	4.92$\times 10^{226}$
**f11**	**Aver.**	8.35$\times 10^{03}$	9.44$\times 10^{-03}$	8.85$\times 10^{01}$	1.09$\times 10^{04}$	1.76$\times 10^{00}$	2.19$\times 10^{00}$	5.08$\times 10^{00}$	1.56$\times 10^{00}$	8.03$\times 10^{-02}$	**3.44$\times 10^{-03}$**	**3.15$\times 10^{-08}$**
	**Stdev.**	1.10$\times 10^{07}$	**4.83$\times 10^{-06}$**	1.89$\times 10^{03}$	3.77$\times 10^{07}$	1.44$\times 10^{00}$	1.32$\times 10^{00}$	8.50$\times 10^{00}$	6.74$\times 10^{-01}$	5.38$\times 10^{-02}$	1.06$\times 10^{-04}$	**1.70$\times 10^{-14}$**
**f12**	**Aver.**	1.99$\times 10^{02}$	9.64$\times 10^{01}$	2.66$\times 10^{02}$	4.02$\times 10^{02}$	4.90$\times 10^{01}$	1.09$\times 10^{01}$	2.70$\times 10^{01}$	2.01$\times 10^{01}$	5.04$\times 10^{-02}$	1.50$\times 10^{00}$	**1.04$\times 10^{-02}$**
	**Stdev.**	6.40$\times 10^{03}$	8.99$\times 10^{02}$	6.66$\times 10^{02}$	5.64$\times 10^{04}$	3.18$\times 10^{03}$	1.38$\times 10^{02}$	2.05$\times 10^{03}$	1.25$\times 10^{01}$	**5.08$\times 10^{-02}$**	1.58$\times 10^{01}$	**1.02$\times 10^{-03}$**
**f13**	**Aver.**	1.27$\times 10^{03}$	1.52$\times 10^{03}$	1.58$\times 10^{03}$	1.05$\times 10^{03}$	5.02$\times 10^{02}$	6.35$\times 10^{02}$	1.21$\times 10^{03}$	3.10$\times 10^{02}$	**1.24$\times 10^{00}$**	6.22$\times 10^{02}$	1.85$\times 10^{01}$
	**Stdev.**	6.42$\times 10^{04}$	3.04$\times 10^{05}$	1.51$\times 10^{04}$	1.68$\times 10^{05}$	2.96$\times 10^{04}$	4.63$\times 10^{04}$	9.37$\times 10^{04}$	4.30$\times 10^{03}$	**2.16$\times 10^{01}$**	5.52$\times 10^{04}$	**2.82$\times 10^{03}$**
**f14**	**Aver.**	7.47$\times 10^{05}$	7.95$\times 10^{02}$	1.58$\times 10^{02}$	5.39$\times 10^{06}$	**2.57$\times 10^{01}$**	6.16$\times 10^{01}$	1.03$\times 10^{02}$	3.24$\times 10^{01}$	1.57$\times 10^{02}$	6.14$\times 10^{01}$	7.55$\times 10^{01}$
	**Stdev.**	3.62$\times 10^{11}$	3.97$\times 10^{06}$	7.22$\times 10^{02}$	4.26$\times 10^{13}$	**5.52$\times 10^{02}$**	3.65$\times 10^{03}$	8.46$\times 10^{03}$	2.53$\times 10^{03}$	1.02$\times 10^{04}$	1.51$\times 10^{03}$	1.87$\times 10^{03}$

**Table 7:** Gaussian-guided SAWSA comparison result data when *D* = 30.

Fun.	Meas.	SAWSA *D* = 30							
		*RDE*1	*GRDE*1	*RDE*2	*GRDE*2	*RDE*3	*GRDE*3	*RDE*4	*GRDE*4
**f1**	**Aver.**	9.30$\times 10^{00}$	3.60$\times 10^{00}$	1.52$\times 10^{01}$	**2.12$\times 10^{00}$**	1.00$\times 10^{01}$	**7.06$\times 10^{-01}$**	1.24$\times 10^{01}$	2.18$\times 10^{00}$
	**Stdev.**	**9.93$\times 10^{-01}$**	1.26$\times 10^{00}$	3.13$\times 10^{00}$	1.36$\times 10^{00}$	6.99$\times 10^{00}$	**5.71$\times 10^{-01}$**	3.24$\times 10^{00}$	2.11$\times 10^{00}$
**f2**	**Aver.**	6.09$\times 10^{-01}$	**1.99$\times 10^{-06}$**	1.47$\times 10^{00}$	2.94$\times 10^{-03}$	3.46$\times 10^{-03}$	**5.34$\times 10^{-04}$**	9.00$\times 10^{-01}$	4.64$\times 10^{-03}$
	**Stdev.**	8.95$\times 10^{-03}$	**7.90$\times 10^{-11}$**	3.03$\times 10^{00}$	4.00$\times 10^{-05}$	1.75$\times 10^{-04}$	**1.79$\times 10^{-06}$**	8.00$\times 10^{-01}$	1.74$\times 10^{-04}$
**f3**	**Aver.**	2.65$\times 10^{-11}$	6.07$\times 10^{-13}$	4.26$\times 10^{-18}$	**1.40$\times 10^{-20}$**	3.14$\times 10^{-12}$	2.51$\times 10^{-14}$	6.48$\times 10^{-15}$	**3.57$\times 10^{-21}$**
	**Stdev.**	9.01$\times 10^{-23}$	9.31$\times 10^{-25}$	9.54$\times 10^{-36}$	**4.48$\times 10^{-40}$**	2.88$\times 10^{-23}$	7.44$\times 10^{-27}$	8.39$\times 10^{-28}$	**1.33$\times 10^{-40}$**
**f4**	**Aver.**	−9.96 $\times 10^{-01}$	−9.97 $\times 10^{-01}$	−1.00 $\times 10^{00}$	−1.00 $\times 10^{00}$	−9.97 $\times 10^{-01}$	−9.97 $\times 10^{-01}$	−1.00 $\times 10^{00}$	−1.00 $\times 10^{00}$
	**Stdev.**	4.59$\times 10^{-07}$	7.05$\times 10^{-07}$	**2.02$\times 10^{-10}$**	9.41$\times 10^{-10}$	4.85$\times 10^{-07}$	6.90$\times 10^{-07}$	**2.24$\times 10^{-10}$**	8.77$\times 10^{-10}$
**f5**	**Aver.**	2.37$\times 10^{00}$	−1.17 $\times 10^{00}$	3.61$\times 10^{00}$	−2.37 $\times 10^{00}$	9.22$\times 10^{-01}$	−1.84 $\times 10^{00}$	−7.95 $\times 10^{-01}$	−2.21 $\times 10^{00}$
	**Stdev.**	1.50$\times 10^{00}$	5.67$\times 10^{-01}$	1.44$\times 10^{00}$	**9.57$\times 10^{-26}$**	2.35$\times 10^{00}$	2.54$\times 10^{-01}$	4.49$\times 10^{-01}$	**1.04$\times 10^{-01}$**
**f6**	**Aver.**	5.86$\times 10^{-01}$	**2.65$\times 10^{-01}$**	1.28$\times 10^{00}$	1.87$\times 10^{00}$	7.45$\times 10^{-01}$	1.19$\times 10^{00}$	**5.07$\times 10^{-01}$**	1.32$\times 10^{00}$
	**Stdev.**	**2.79$\times 10^{-01}$**	**6.92$\times 10^{-05}$**	3.17$\times 10^{00}$	1.86$\times 10^{00}$	8.33$\times 10^{-01}$	9.57$\times 10^{-01}$	2.96$\times 10^{-01}$	3.54$\times 10^{00}$
**f7**	**Aver.**	2.85$\times 10^{-11}$	7.01$\times 10^{-13}$	7.14$\times 10^{-18}$	**2.19$\times 10^{-20}$**	2.31$\times 10^{-12}$	1.93$\times 10^{-14}$	1.94$\times 10^{-19}$	**1.45$\times 10^{-21}$**
	**Stdev.**	1.73$\times 10^{-22}$	1.73$\times 10^{-24}$	8.83$\times 10^{-35}$	**2.66$\times 10^{-39}$**	1.20$\times 10^{-23}$	2.81$\times 10^{-27}$	2.14$\times 10^{-37}$	**7.08$\times 10^{-42}$**
**f8**	**Aver.**	9.88$\times 10^{-05}$	9.05$\times 10^{-02}$	1.59$\times 10^{-01}$	**8.98$\times 10^{-09}$**	6.63$\times 10^{-01}$	1.39$\times 10^{-07}$	2.30$\times 10^{-01}$	**1.31$\times 10^{-09}$**
	**Stdev.**	2.99$\times 10^{-10}$	7.77$\times 10^{-02}$	1.16$\times 10^{-01}$	**8.31$\times 10^{-17}$**	1.06$\times 10^{00}$	3.69$\times 10^{-13}$	2.62$\times 10^{-01}$	**2.95$\times 10^{-18}$**
**f9**	**Aver.**	−2.59 $\times 10^{01}$	−2.95 $\times 10^{01}$	−2.50 $\times 10^{01}$	−2.92 $\times 10^{01}$	−2.92 $\times 10^{01}$	−2.96 $\times 10^{01}$	−2.56 $\times 10^{01}$	−2.90 $\times 10^{01}$
	**Stdev.**	3.48$\times 10^{-01}$	**9.69$\times 10^{-03}$**	8.58$\times 10^{-01}$	3.17$\times 10^{-02}$	9.18$\times 10^{-02}$	**1.09$\times 10^{-03}$**	1.74$\times 10^{00}$	8.78$\times 10^{-02}$
**f10**	**Aver.**	4.60$\times 10^{51}$	3.18$\times 10^{51}$	**7.05$\times 10^{48}$**	**7.28$\times 10^{48}$**	8.05$\times 10^{51}$	5.61$\times 10^{51}$	2.45$\times 10^{49}$	1.01$\times 10^{49}$
	**Stdev.**	4.22$\times 10^{103}$	1.79$\times 10^{103}$	**1.66$\times 10^{98}$**	**1.49$\times 10^{98}$**	3.81$\times 10^{104}$	1.18$\times 10^{104}$	1.04$\times 10^{99}$	1.77$\times 10^{98}$
**f11**	**Aver.**	1.40$\times 10^{-01}$	**2.77$\times 10^{-30}$**	7.65$\times 10^{-03}$	2.09$\times 10^{-03}$	**1.65$\times 10^{-13}$**	7.80$\times 10^{-05}$	7.05$\times 10^{-05}$	5.70$\times 10^{-04}$
	**Stdev.**	2.16$\times 10^{-03}$	**6.80$\times 10^{-59}$**	1.92$\times 10^{-04}$	2.49$\times 10^{-06}$	**5.47$\times 10^{-25}$**	7.53$\times 10^{-08}$	1.02$\times 10^{-08}$	1.52$\times 10^{-06}$
**f12**	**Aver.**	5.04$\times 10^{00}$	3.63$\times 10^{-02}$	3.05$\times 10^{01}$	**5.18$\times 10^{-03}$**	7.39$\times 10^{-01}$	**9.88$\times 10^{-09}$**	4.59$\times 10^{00}$	1.04$\times 10^{-02}$
	**Stdev.**	3.34$\times 10^{00}$	1.05$\times 10^{-02}$	1.32$\times 10^{02}$	**5.37$\times 10^{-04}$**	3.57$\times 10^{00}$	**6.32$\times 10^{-16}$**	4.51$\times 10^{01}$	1.02$\times 10^{-03}$
**f13**	**Aver.**	1.35$\times 10^{02}$	**4.48$\times 10^{-01}$**	3.88$\times 10^{02}$	1.47$\times 10^{01}$	2.11$\times 10^{02}$	**5.00$\times 10^{-02}$**	1.78$\times 10^{02}$	2.06$\times 10^{01}$
	**Stdev.**	2.17$\times 10^{03}$	**1.40$\times 10^{00}$**	4.78$\times 10^{03}$	5.63$\times 10^{01}$	1.21$\times 10^{04}$	**4.97$\times 10^{-02}$**	4.00$\times 10^{03}$	8.82$\times 10^{01}$
**f14**	**Aver.**	**1.83$\times 10^{01}$**	4.25$\times 10^{01}$	4.62$\times 10^{01}$	5.55$\times 10^{01}$	**2.07$\times 10^{01}$**	5.01$\times 10^{01}$	4.63$\times 10^{01}$	4.04$\times 10^{01}$
	**Stdev.**	**6.48$\times 10^{02}$**	1.45$\times 10^{03}$	1.15$\times 10^{03}$	5.43$\times 10^{03}$	**7.11$\times 10^{02}$**	2.26$\times 10^{03}$	1.56$\times 10^{03}$	2.60$\times 10^{03}$

**Table 8:** Gaussian-guided SAWSA comparison result data when *D* = 50.

Fun.	Meas.	SAWSA *D* = 50							
		*RDE*1	*GRDE*1	*RDE*2	*GRDE*2	*RDE*3	*GRDE*3	*RDE*4	*GRDE*4
**f1**	**Aver.**	1.72$\times 10^{01}$	1.20$\times 10^{01}$	5.15$\times 10^{00}$	**1.32$\times 10^{00}$**	1.22$\times 10^{01}$	1.47$\times 10^{01}$	6.21$\times 10^{00}$	**5.10$\times 10^{00}$**
	**Stdev.**	1.79$\times 10^{00}$	**4.93$\times 10^{-01}$**	3.64$\times 10^{00}$	**8.02$\times 10^{-01}$**	3.62$\times 10^{00}$	1.29$\times 10^{00}$	4.56$\times 10^{00}$	1.30$\times 10^{01}$
**f2**	**Aver.**	2.59$\times 10^{00}$	6.12$\times 10^{00}$	4.93$\times 10^{-02}$	**4.09$\times 10^{-02}$**	2.67$\times 10^{-01}$	5.06$\times 10^{00}$	**4.27$\times 10^{-02}$**	7.12$\times 10^{-02}$
	**Stdev.**	1.01$\times 10^{00}$	3.80$\times 10^{01}$	9.06$\times 10^{-03}$	**3.72$\times 10^{-03}$**	3.18$\times 10^{-01}$	3.17$\times 10^{00}$	3.50$\times 10^{-02}$	**5.61$\times 10^{-03}$**
**f3**	**Aver.**	1.08$\times 10^{-09}$	1.28$\times 10^{-09}$	2.41$\times 10^{-12}$	**1.69$\times 10^{-12}$**	9.25$\times 10^{-10}$	5.01$\times 10^{-09}$	3.17$\times 10^{-10}$	**1.29$\times 10^{-19}$**
	**Stdev.**	2.86$\times 10^{-19}$	7.54$\times 10^{-18}$	9.79$\times 10^{-23}$	**6.04$\times 10^{-24}$**	6.34$\times 10^{-18}$	2.38$\times 10^{-16}$	4.34$\times 10^{-19}$	**1.58$\times 10^{-38}$**
**f4**	**Aver.**	−9.88$\times 10^{-01}$	−9.96$\times 10^{-01}$	−9.89$\times 10^{-01}$	−1.00$\times 10^{00}$	−9.90$\times 10^{-01}$	**−1.00$\times 10^{00}$**	−9.89$\times 10^{-01}$	**−1.00$\times 10^{00}$**
	**Stdev.**	4.22$\times 10^{-06}$	5.50$\times 10^{-05}$	2.76$\times 10^{-06}$	**2.72$\times 10^{-09}$**	3.79$\times 10^{-06}$	**3.04$\times 10^{-10}$**	2.61$\times 10^{-06}$	3.99$\times 10^{-09}$
**f5**	**Aver.**	7.87$\times 10^{00}$	1.05$\times 10^{01}$	−1.89$\times 10^{00}$	**−3.47$\times 10^{00}$**	3.73$\times 10^{00}$	2.66$\times 10^{-01}$	−5.62$\times 10^{-01}$	**−3.03$\times 10^{00}$**
	**Stdev.**	4.83$\times 10^{00}$	2.82$\times 10^{00}$	8.01$\times 10^{-01}$	**1.15$\times 10^{-01}$**	8.86$\times 10^{00}$	3.29$\times 10^{00}$	1.59$\times 10^{00}$	**3.21$\times 10^{-01}$**
**f6**	**Aver.**	1.42$\times 10^{00}$	2.95$\times 10^{00}$	6.61$\times 10^{00}$	6.48$\times 10^{00}$	1.48$\times 10^{00}$	**6.69$\times 10^{-01}$**	**8.28$\times 10^{-01}$**	3.77$\times 10^{00}$
	**Stdev.**	**4.32$\times 10^{-01}$**	1.11$\times 10^{01}$	1.93$\times 10^{01}$	1.07$\times 10^{02}$	2.52$\times 10^{00}$	**6.77$\times 10^{-01}$**	1.29$\times 10^{00}$	1.19$\times 10^{01}$
**f7**	**Aver.**	1.21$\times 10^{-09}$	1.75$\times 10^{-09}$	1.32$\times 10^{-12}$	**1.09$\times 10^{-17}$**	1.97$\times 10^{-10}$	2.45$\times 10^{-09}$	2.13$\times 10^{-09}$	**8.39$\times 10^{-20}$**
	**Stdev.**	3.46$\times 10^{-19}$	4.78$\times 10^{-18}$	3.08$\times 10^{-24}$	**2.67$\times 10^{-34}$**	2.66$\times 10^{-19}$	4.81$\times 10^{-17}$	8.12$\times 10^{-17}$	**6.46$\times 10^{-39}$**
**f8**	**Aver.**	1.36$\times 10^{-01}$	2.52$\times 10^{01}$	8.59$\times 10^{-07}$	**9.72$\times 10^{-08}$**	5.68$\times 10^{-01}$	3.29$\times 10^{-01}$	1.85$\times 10^{-01}$	**1.10$\times 10^{-08}$**
	**Stdev.**	3.67$\times 10^{-01}$	2.13$\times 10^{02}$	1.65$\times 10^{-12}$	**4.86$\times 10^{-15}$**	1.82$\times 10^{00}$	5.98$\times 10^{-01}$	2.39$\times 10^{-01}$	**7.24$\times 10^{-17}$**
**f9**	**Aver.**	−3.94$\times 10^{01}$	−3.92$\times 10^{01}$	−4.71$\times 10^{01}$	**−4.86$\times 10^{01}$**	−4.81$\times 10^{01}$	−4.00$\times 10^{01}$	−4.67$\times 10^{01}$	**−4.86$\times 10^{01}$**
	**Stdev.**	5.30$\times 10^{-01}$	4.85$\times 10^{00}$	**1.90$\times 10^{-01}$**	2.05$\times 10^{-01}$	2.11$\times 10^{-01}$	3.47$\times 10^{00}$	2.46$\times 10^{-01}$	**1.49$\times 10^{-01}$**
**f10**	**Aver.**	7.82$\times 10^{115}$	**5.62$\times 10^{112}$**	3.60$\times 10^{115}$	9.41$\times 10^{112}$	3.47$\times 10^{115}$	1.01$\times 10^{113}$	1.07$\times 10^{116}$	**7.39$\times 10^{112}$**
	**Stdev.**	2.19$\times 10^{226}$	**3.82$\times 10^{225}$**	4.84$\times 10^{226}$	3.08$\times 10^{226}$	2.19$\times 10^{226}$	2.18$\times 10^{226}$	4.92$\times 10^{226}$	**1.20$\times 10^{226}$**
**f11**	**Aver.**	1.56$\times 10^{00}$	5.17$\times 10^{-01}$	8.03$\times 10^{-02}$	5.54$\times 10^{-02}$	3.44$\times 10^{-03}$	**3.15$\times 10^{-08}$**	**8.48$\times 10^{-04}$**	2.66$\times 10^{-02}$
	**Stdev.**	6.74$\times 10^{-01}$	1.39$\times 10^{-01}$	5.38$\times 10^{-02}$	1.03$\times 10^{-02}$	1.06$\times 10^{-04}$	**1.70$\times 10^{-14}$**	**1.30$\times 10^{-06}$**	3.65$\times 10^{-03}$
**f12**	**Aver.**	2.01$\times 10^{01}$	1.04$\times 10^{02}$	5.04$\times 10^{-02}$	**1.04$\times 10^{-02}$**	1.50$\times 10^{00}$	2.48$\times 10^{01}$	4.66$\times 10^{-02}$	**1.04$\times 10^{-02}$**
	**Stdev.**	1.25$\times 10^{01}$	3.16$\times 10^{03}$	5.08$\times 10^{-02}$	**2.15$\times 10^{-03}$**	1.58$\times 10^{01}$	2.94$\times 10^{02}$	1.07$\times 10^{-02}$	**1.02$\times 10^{-03}$**
**f13**	**Aver.**	3.10$\times 10^{02}$	8.46$\times 10^{02}$	4.84$\times 10^{01}$	**1.24$\times 10^{00}$**	6.22$\times 10^{02}$	5.42$\times 10^{02}$	4.95$\times 10^{01}$	**1.85$\times 10^{01}$**
	**Stdev.**	4.30$\times 10^{03}$	8.49$\times 10^{03}$	**2.67$\times 10^{02}$**	**2.16$\times 10^{01}$**	5.52$\times 10^{04}$	1.46$\times 10^{04}$	2.82$\times 10^{03}$	5.36$\times 10^{02}$
**f14**	**Aver.**	9.67$\times 10^{01}$	**3.24$\times 10^{01}$**	1.57$\times 10^{02}$	6.14$\times 10^{01}$	1.30$\times 10^{02}$	**5.93$\times 10^{01}$**	7.55$\times 10^{01}$	1.37$\times 10^{02}$
	**Stdev.**	9.73$\times 10^{03}$	2.53$\times 10^{03}$	1.02$\times 10^{04}$	**1.51$\times 10^{03}$**	6.80$\times 10^{03}$	**1.57$\times 10^{03}$**	1.87$\times 10^{03}$	4.85$\times 10^{03}$

**Table 9:** *h* and *p*-value for the statistical test with WSA as the control method.

***D* = 30**	***DE*1**		***DE*2**		***DE*3**		***RDE*1**		***RDE*2**		***RDE*3**		***RDE*4**		***GRDE*1**		***GRDE*2**		***GRDE*3**		***GRDE*4**	
	***h***	***p***	***h***	***p***	***h***	***p***	***h***	***p***	***h***	***p***	***h***	***p***	***h***	***p***	***h***	***p***	***h***	***p***	***h***	***p***	***h***	***p***
**f1**	1	8.02$\times 10^{-36}$	1	2.00$\times 10^{-36}$	1	1.31$\times 10^{-27}$	1	1.02$\times 10^{-39}$	1	3.10$\times 10^{-17}$	1	1.99$\times 10^{-44}$	1	1.07$\times 10^{-45}$	1	2.53$\times 10^{-41}$	1	9.25$\times 10^{-26}$	1	1.35$\times 10^{-72}$	1	1.31$\times 10^{-40}$
**f2**	1	1.69$\times 10^{-56}$	1	2.30$\times 10^{-63}$	1	1.14$\times 10^{-59}$	1	1.48$\times 10^{-62}$	1	1.75$\times 10^{-71}$	1	5.04$\times 10^{-66}$	1	6.23$\times 10^{-58}$	1	6.82$\times 10^{-43}$	1	1.17$\times 10^{-77}$	1	2.68$\times 10^{-77}$	1	1.50$\times 10^{-151}$
**f3**	1	2.03$\times 10^{-208}$	1	2.03$\times 10^{-208}$	1	2.03$\times 10^{-208}$	1	2.03$\times 10^{-208}$	1	2.03$\times 10^{-208}$	1	2.03$\times 10^{-208}$	1	2.03$\times 10^{-208}$	1	2.03$\times 10^{-208}$	1	2.03$\times 10^{-208}$	1	2.03$\times 10^{-208}$	1	0.00$\times 10^{00}$
**f4**	1	1.37$\times 10^{-124}$	1	7.95$\times 10^{-125}$	1	5.99$\times 10^{-124}$	1	1.41$\times 10^{-124}$	1	8.09$\times 10^{-125}$	1	6.01$\times 10^{-125}$	1	4.22$\times 10^{-124}$	1	3.00$\times 10^{-120}$	1	1.75$\times 10^{-130}$	1	3.72$\times 10^{-103}$	1	5.69$\times 10^{-88}$
**f5**	1	1.76$\times 10^{-42}$	1	3.88$\times 10^{-39}$	1	6.02$\times 10^{-46}$	1	2.60$\times 10^{-55}$	1	5.42$\times 10^{-76}$	1	1.96$\times 10^{-76}$	1	4.87$\times 10^{-60}$	1	5.96$\times 10^{-45}$	1	1.05$\times 10^{-70}$	1	1.96$\times 10^{-78}$	1	7.76$\times 10^{-142}$
**f6**	1	1.07$\times 10^{-15}$	1	2.91$\times 10^{-10}$	1	4.87$\times 10^{-10}$	1	2.84$\times 10^{-14}$	1	4.34$\times 10^{-31}$	1	5.32$\times 10^{-32}$	1	5.42$\times 10^{-35}$	1	2.88$\times 10^{-32}$	0	2.02$\times 10^{-01}$	1	5.86$\times 10^{-18}$	0	9.16$\times 10^{-01}$
**f7**	1	7.09$\times 10^{-186}$	1	7.09$\times 10^{-186}$	1	7.09$\times 10^{-186}$	1	7.09$\times 10^{-186}$	1	7.09$\times 10^{-186}$	1	7.09$\times 10^{-186}$	1	7.09$\times 10^{-186}$	1	7.09$\times 10^{-186}$	1	7.09$\times 10^{-186}$	1	7.09$\times 10^{-186}$	1	0.00$\times 10^{00}$
**f8**	1	9.35$\times 10^{-45}$	1	3.60$\times 10^{-38}$	1	3.75$\times 10^{-42}$	1	1.27$\times 10^{-36}$	1	2.22$\times 10^{-69}$	1	6.62$\times 10^{-73}$	1	5.89$\times 10^{-59}$	1	1.07$\times 10^{-40}$	1	1.35$\times 10^{-75}$	1	3.53$\times 10^{-77}$	1	4.59$\times 10^{-150}$
**f9**	1	9.53$\times 10^{-82}$	1	8.29$\times 10^{-82}$	1	5.97$\times 10^{-67}$	1	5.37$\times 10^{-76}$	1	6.33$\times 10^{-88}$	1	6.11$\times 10^{-98}$	1	2.08$\times 10^{-94}$	1	2.18$\times 10^{-58}$	1	3.69$\times 10^{-75}$	1	3.81$\times 10^{-76}$	1	9.80$\times 10^{-81}$
**f10**	0	9.40$\times 10^{-02}$	1	1.81$\times 10^{-02}$	1	1.49$\times 10^{-02}$	0	9.15$\times 10^{-01}$	0	8.93$\times 10^{-02}$	1	1.91$\times 10^{-02}$	1	1.62$\times 10^{-02}$	1	9.75$\times 10^{-03}$	1	9.74$\times 10^{-03}$	1	9.74$\times 10^{-03}$	1	7.87$\times 10^{-06}$
**f11**	1	5.65$\times 10^{-08}$	1	7.67$\times 10^{-20}$	1	2.20$\times 10^{-06}$	1	1.90$\times 10^{-03}$	1	9.16$\times 10^{-21}$	1	9.13$\times 10^{-21}$	1	9.15$\times 10^{-21}$	1	1.09$\times 10^{-29}$	1	1.39$\times 10^{-20}$	1	1.66$\times 10^{-20}$	1	3.90$\times 10^{-51}$
**f12**	1	2.49$\times 10^{-42}$	1	9.71$\times 10^{-38}$	1	5.12$\times 10^{-35}$	1	5.23$\times 10^{-35}$	1	1.61$\times 10^{-36}$	1	2.61$\times 10^{-48}$	1	3.09$\times 10^{-49}$	1	7.61$\times 10^{-45}$	1	2.21$\times 10^{-66}$	1	1.49$\times 10^{-78}$	1	1.66$\times 10^{-132}$
**f13**	1	8.46$\times 10^{-68}$	1	2.39$\times 10^{-45}$	1	3.15$\times 10^{-50}$	1	1.28$\times 10^{-52}$	1	8.80$\times 10^{-97}$	1	6.28$\times 10^{-97}$	1	1.37$\times 10^{-70}$	1	5.58$\times 10^{-55}$	1	1.44$\times 10^{-96}$	1	2.44$\times 10^{-97}$	1	3.21$\times 10^{-155}$
**f14**	1	2.79$\times 10^{-18}$	1	1.94$\times 10^{-06}$	1	4.93$\times 10^{-14}$	1	7.06$\times 10^{-20}$	0	1.46$\times 10^{-01}$	1	7.86$\times 10^{-04}$	1	2.37$\times 10^{-04}$	1	1.47$\times 10^{-31}$	1	2.18$\times 10^{-05}$	1	3.21$\times 10^{-06}$	1	3.67$\times 10^{-03}$
***D* = 50**	***DE*1**		***DE*2**		***DE*3**		***RDE*1**		***RDE*2**		***RDE*3**		***RDE*4**		***GRDE*1**		***GRDE*2**		***GRDE*3**		***GRDE*4**	
	***h***	***p***	***h***	***p***	***h***	***p***	***h***	***p***	***h***	***p***	***h***	***p***	***h***	***p***	***h***	***p***	***h***	***p***	***h***	***p***	***h***	***p***
**f1**	1	4.20$\times 10^{-35}$	1	6.44$\times 10^{-28}$	1	4.12$\times 10^{-35}$	1	2.73$\times 10^{-36}$	1	4.00$\times 10^{-11}$	1	1.20$\times 10^{-45}$	1	5.52$\times 10^{-33}$	1	4.54$\times 10^{-47}$	1	5.75$\times 10^{-17}$	1	8.03$\times 10^{-75}$	1	4.34$\times 10^{-27}$
**f2**	1	9.99$\times 10^{-43}$	1	2.70$\times 10^{-48}$	1	1.79$\times 10^{-55}$	1	2.80$\times 10^{-52}$	1	4.53$\times 10^{-66}$	1	1.43$\times 10^{-63}$	1	7.30$\times 10^{-44}$	1	1.95$\times 10^{-36}$	1	5.95$\times 10^{-69}$	1	1.23$\times 10^{-69}$	1	1.60$\times 10^{-133}$
**f3**	1	1.82$\times 10^{-188}$	1	1.80$\times 10^{-188}$	1	1.81$\times 10^{-188}$	1	1.82$\times 10^{-188}$	1	1.80$\times 10^{-188}$	1	1.80$\times 10^{-188}$	1	1.80$\times 10^{-188}$	1	1.88$\times 10^{-188}$	1	1.80$\times 10^{-188}$	1	1.80$\times 10^{-188}$	1	0.00$\times 10^{00}$
**f4**	1	5.81$\times 10^{-125}$	1	1.51$\times 10^{-127}$	1	3.78$\times 10^{-132}$	1	5.17$\times 10^{-131}$	1	8.09$\times 10^{-127}$	1	1.54$\times 10^{-126}$	1	6.80$\times 10^{-126}$	1	2.86$\times 10^{-107}$	1	7.01$\times 10^{-136}$	1	4.82$\times 10^{-123}$	1	2.42$\times 10^{-94}$
**f5**	1	1.07$\times 10^{-22}$	1	6.12$\times 10^{-37}$	1	3.06$\times 10^{-47}$	1	1.70$\times 10^{-46}$	1	8.79$\times 10^{-78}$	1	1.17$\times 10^{-80}$	1	1.02$\times 10^{-60}$	1	8.69$\times 10^{-45}$	1	1.33$\times 10^{-70}$	1	4.51$\times 10^{-83}$	1	2.26$\times 10^{-135}$
**f6**	1	4.12$\times 10^{-22}$	0	9.42$\times 10^{-01}$	1	1.09$\times 10^{-18}$	1	2.17$\times 10^{-33}$	0	3.83$\times 10^{-01}$	1	2.65$\times 10^{-20}$	1	1.14$\times 10^{-21}$	1	6.92$\times 10^{-44}$	1	3.08$\times 10^{-05}$	0	5.81$\times 10^{-02}$	1	1.06$\times 10^{-04}$
**f7**	1	3.11$\times 10^{-178}$	1	3.07$\times 10^{-178}$	1	3.09$\times 10^{-178}$	1	3.09$\times 10^{-178}$	1	3.07$\times 10^{-178}$	1	3.07$\times 10^{-178}$	1	3.07$\times 10^{-178}$	1	3.15$\times 10^{-178}$	1	3.07$\times 10^{-178}$	1	3.07$\times 10^{-178}$	1	0.00$\times 10^{00}$
**f8**	1	1.22$\times 10^{-36}$	1	1.05$\times 10^{-37}$	1	5.78$\times 10^{-36}$	1	6.06$\times 10^{-49}$	1	2.13$\times 10^{-40}$	1	3.12$\times 10^{-57}$	1	7.67$\times 10^{-46}$	1	1.56$\times 10^{-38}$	1	2.48$\times 10^{-66}$	1	2.41$\times 10^{-82}$	1	2.02$\times 10^{-119}$
**f9**	1	4.12$\times 10^{-74}$	1	2.40$\times 10^{-71}$	1	1.59$\times 10^{-71}$	1	3.13$\times 10^{-76}$	1	3.58$\times 10^{-83}$	1	1.47$\times 10^{-90}$	1	2.49$\times 10^{-83}$	1	1.13$\times 10^{-57}$	1	5.19$\times 10^{-54}$	1	2.55$\times 10^{-57}$	1	1.29$\times 10^{-64}$
**f10**	0	3.20$\times 10^{-01}$	0	3.20$\times 10^{-01}$	0	3.20$\times 10^{-01}$	0	3.20$\times 10^{-01}$	0	3.20$\times 10^{-01}$	0	3.20$\times 10^{-01}$	0	3.20$\times 10^{-01}$	0	1.76$\times 10^{-01}$	0	3.20$\times 10^{-01}$	0	3.12$\times 10^{-01}$	0	7.95$\times 10^{-01}$
**f11**	1	4.76$\times 10^{-23}$	1	3.98$\times 10^{-29}$	1	2.41$\times 10^{-16}$	1	1.02$\times 10^{-28}$	1	2.53$\times 10^{-31}$	1	2.13$\times 10^{-31}$	1	2.07$\times 10^{-31}$	1	3.70$\times 10^{-49}$	1	2.05$\times 10^{-29}$	1	1.36$\times 10^{-30}$	1	1.18$\times 10^{-70}$
**f12**	1	1.51$\times 10^{-35}$	1	3.43$\times 10^{-27}$	1	1.75$\times 10^{-18}$	1	2.06$\times 10^{-29}$	1	2.90$\times 10^{-22}$	1	1.64$\times 10^{-45}$	1	1.87$\times 10^{-27}$	1	5.47$\times 10^{-37}$	1	1.42$\times 10^{-52}$	1	1.26$\times 10^{-85}$	1	1.38$\times 10^{-102}$
**f13**	1	2.62$\times 10^{-41}$	1	4.16$\times 10^{-42}$	1	4.73$\times 10^{-50}$	1	3.14$\times 10^{-72}$	1	6.89$\times 10^{-99}$	1	1.08$\times 10^{-98}$	1	1.06$\times 10^{-73}$	1	4.37$\times 10^{-55}$	1	1.21$\times 10^{-97}$	1	1.30$\times 10^{-99}$	1	1.84$\times 10^{-136}$
**f14**	1	3.62$\times 10^{-34}$	1	3.04$\times 10^{-04}$	1	1.47$\times 10^{-22}$	1	4.39$\times 10^{-33}$	1	8.42$\times 10^{-04}$	1	8.91$\times 10^{-05}$	0	5.79$\times 10^{-01}$	1	9.15$\times 10^{-51}$	1	2.23$\times 10^{-08}$	1	1.82$\times 10^{-04}$	1	7.67$\times 10^{-05}$

**Table 10:** CPU time comparison with 20 search agents.

***D* = 30**	**PSO**	**BA**	**HSABA**	**WSA**	***DE*1**	***DE*2**	***DE*3**	***RDE*1**	***RDE*2**	***RDE*3**	***RDE*4**	***GRDE*1**	***GRDE*2**	***GRDE*3**	***GRDE*4**
					**SAWSA**	**SAWSA**	**SAWSA**	**SAWSA**	**SAWSA**	**SAWSA**	**SAWSA**	**SAWSA**	**SAWSA**	**SAWSA**	**SAWSA**
**f1**	4.40	1.92	9.26	8.19	11.24	11.44	11.44	11.98	12.61	12.67	12.55	13.12	13.79	13.70	13.49
**f2**	4.32	2.02	9.38	7.80	10.94	11.17	11.04	11.64	12.05	12.30	11.73	12.85	12.78	12.84	12.59
**f3**	7.16	2.51	13.77	10.68	14.81	15.10	15.54	15.87	16.57	16.43	16.83	16.41	16.65	16.54	16.74
**f4**	7.13	2.76	13.72	10.15	14.60	14.55	14.44	15.54	15.35	15.37	15.52	15.96	15.97	16.24	15.83
**f5**	5.06	2.16	10.62	8.40	11.76	11.82	11.72	12.47	12.46	12.58	12.54	13.54	13.69	13.52	13.53
**f6**	3.88	1.88	8.49	7.84	10.63	10.61	11.17	11.25	12.02	12.09	11.95	12.30	12.28	12.59	12.18
**f7**	6.75	2.44	13.08	10.26	14.39	14.49	15.28	15.29	16.51	15.99	16.12	15.82	15.97	16.16	16.12
**f8**	6.30	2.57	12.65	9.45	13.14	13.30	14.09	13.79	14.84	14.85	14.61	15.28	15.61	15.44	15.62
**f9**	7.28	2.72	14.36	10.54	14.67	14.73	15.04	15.51	15.46	15.77	15.61	16.49	16.73	16.59	16.62
**f10**	10.00	3.42	18.77	12.45	17.05	17.27	17.14	17.84	18.04	17.99	18.12	19.89	20.14	19.94	20.06
**f11**	3.43	1.77	7.82	7.47	10.12	10.30	10.51	10.93	11.75	11.73	11.50	11.66	12.05	11.81	11.87
**f12**	9.76	3.56	19.12	12.58	16.15	15.64	17.47	16.38	16.81	16.83	16.41	19.50	17.55	17.99	17.60
**f13**	4.16	1.90	9.19	7.73	11.08	10.88	11.17	11.54	12.18	12.11	12.02	12.76	12.73	12.75	12.79
**f14**	3.77	1.88	8.28	7.66	10.44	10.55	10.88	11.09	11.97	11.91	11.75	12.10	12.48	12.20	12.08
***D* = 50**	**PSO**	**BA**	**HSABA**	**WSA**	***DE***	***DE***	***DE*3**	***RDE*1**	***RDE*2**	***RDE*3**	***RDE*4**	***GRDE*1**	***GRDE*2**	***GRDE*3**	***GRDE*4**
					**SAWSA**	**SAWSA**	**SAWSA**	**SAWSA**	**SAWSA**	**SAWSA**	**SAWSA**	**SAWSA**	**SAWSA**	**SAWSA**	**SAWSA**
**f1**	5.09	2.25	10.75	9.56	12.82	13.06	13.43	13.56	14.33	13.49	13.91	14.90	14.89	14.86	15.03
**f2**	5.05	2.36	11.08	9.22	12.59	12.46	12.59	13.45	13.76	13.44	13.14	14.40	14.23	14.52	14.44
**f3**	9.82	3.21	18.21	13.52	18.45	18.44	19.31	19.39	20.59	20.80	20.55	20.29	20.61	20.40	20.66
**f4**	9.54	3.42	18.58	13.26	18.05	18.05	18.00	18.89	18.96	18.88	18.96	19.71	19.86	19.83	19.79
**f5**	6.21	2.58	12.84	9.97	13.69	13.58	13.72	14.25	14.29	14.33	14.53	15.70	15.95	15.69	15.72
**f6**	4.31	2.27	9.60	8.76	11.54	11.69	12.28	12.21	13.20	13.32	12.99	13.52	13.93	13.70	13.56
**f7**	9.15	3.05	17.07	12.87	17.83	17.72	18.56	18.58	19.82	19.47	19.51	19.65	19.73	19.39	19.64
**f8**	8.14	3.27	16.46	11.70	16.02	15.88	16.98	16.50	18.08	18.05	17.59	18.57	18.70	18.73	18.61
**f9**	10.34	3.62	19.72	13.77	18.57	18.43	18.51	19.05	19.33	19.17	19.21	20.89	20.75	20.76	20.64
**f10**	10.23	3.84	19.77	13.69	18.14	18.12	17.98	18.76	19.02	18.95	19.06	21.17	21.30	20.96	21.26
**f11**	3.76	2.03	8.82	8.41	11.38	11.21	11.55	12.02	12.65	12.70	12.43	12.95	13.15	12.88	13.17
**f12**	13.46	4.81	26.55	17.04	21.09	20.65	20.80	21.36	21.46	21.44	21.11	25.42	22.31	23.89	22.22
**f13**	5.09	2.26	11.01	9.29	12.32	12.44	13.03	13.03	13.48	14.08	13.39	14.48	14.47	14.35	14.41
**f14**	4.31	2.31	9.53	8.60	11.55	11.53	12.13	12.24	13.16	13.30	12.98	13.58	13.85	13.76	13.31

## Appendix A

Processor: Intel(R) Core(TM) i7-4790 CPU @3.60 GHz

Installed memory (RAM): 8.00 GB

System: Windows 7 Enterprise Service Pack 1 64-bit

Development environment: MATLAB R2014a 64-bit
